# *CLEC19A* overexpression inhibits tumor cell proliferation/migration and promotes apoptosis concomitant suppression of *PI3K/AKT/NF-κB* signaling pathway in glioblastoma multiforme

**DOI:** 10.1186/s12885-023-11755-9

**Published:** 2024-01-02

**Authors:** Fatemeh Mohajerani, Zahra Moazezi Tehrankhah, Saeid Rahmani, Nastaran Afsordeh, Sajad Shafiee, Mohammad Hossein Pourgholami, Bahram M. Soltani, Majid Sadeghizadeh

**Affiliations:** 1 Department of Genetics, Faculty of Biological Sciences, Tarbiat Modares University, Jalal AleAhmad Highway, Tehran, Iran; 2 School of Computer Science, Institute for Research in Fundamental Sciences (IPM), Tehran, Iran; 3 Department of Physiology, Faculty of Medical Sciences, Tarbiat Modares University, Tehran, Iran; 4 Department of Neurosurgery, Mazandaran University of Medical Sciences, Sari, Iran

**Keywords:** *CLEC19A*, C-type lectin, Glioblastoma, PI3K/AKT/NF-κB pathway, Tumor suppressor gene

## Abstract

**Background:**

GBM is the most frequent malignant primary brain tumor in humans. The *CLEC19A* is a member of the C-type lectin family, which has a high expression in brain tissue. Herein, we sought to carry out an in-depth analysis to pinpoint the role of *CLEC19A* expression in GBM.

**Methods:**

To determine the localization of CLEC19A, this protein was detected using Western blot, Immunocytochemistry/Immunofluorescence, and confocal microscopy imaging. CLEC19A expression in glioma cells and tissues was evaluated by qRT-PCR. Cell viability, proliferation, migration, and apoptosis were examined through MTT assay, CFSE assay, colony formation, wound healing assay, transwell test, and flow cytometry respectively after CLEC19A overexpression. The effect of CLEC19A overexpression on the *PI3K/AKT/NF-κB* signaling pathway was investigated using Western blot. An in vivo experiment substantiated the in vitro results using the glioblastoma rat models.

**Results:**

Our *in-silico* analysis using TCGA data and measuring *CLEC19A* expression level by qRT-PCR determined significantly lower expression of *CLEC19A* in human glioma tissues compared to healthy brain tissues. By employment of ICC/IF, confocal microscopy imaging, and Western blot we could show that CLEC19A is plausibly a secreted protein. Results obtained from several in vitro readouts showed that *CLEC19A* overexpression in U87 and C6 glioma cell lines is associated with the inhibition of cell proliferation, viability, and migration. Further, qRT-PCR and Western blot analysis showed *CLEC19A* overexpression could reduce the expression levels of PI3K, VEGFα, MMP2, and NF-κB and increase *PTEN*, *TIMP3*, *RECK*, and *PDCD4* expression levels in glioma cell lines. Furthermore, flow cytometry results revealed that *CLEC19A* overexpression was associated with significant cell cycle arrest and promotion of apoptosis in glioma cell lines. Interestingly, using a glioma rat model we could substantiate that *CLEC19A* overexpression suppresses glioma tumor growth.

**Conclusions:**

To our knowledge, this is the first report providing *in-silico*, molecular, cellular, and in vivo evidences on the role of *CLEC19A* as a putative tumor suppressor gene in GBM. These results enhance our understanding of the role of *CLEC19A* in glioma and warrant further exploration of *CLEC19A* as a potential therapeutic target for GBM.

**Supplementary Information:**

The online version contains supplementary material available at 10.1186/s12885-023-11755-9.

## Background

Glioblastoma multiforme (GBM) is the most frequent malignant primary brain tumor with the average diagnosis at around the age of 60 years [1]. GBM is defined as grade IV glioblastoma by the World Health Organization (WHO), and the median survival of patients is approximately 15 months [2]. The migrating glioma cells from the tumor core in the brain are common, which can complicate surgery, radiotherapy, and chemotherapy [3]. In recent years, many studies have investigated the molecular pathways involved in GBM cancer. In the current study sought to carry out an in-depth analysis of molecular pathways that of potentially important in GBM.

It has been established that the promotion of the PI3K/AKT signaling pathway has an important role in carcinogenesis and has disturbed various human cancers [4]. Also, it has been reported that the overactivity of the PI3K/AKT/NF-κB signaling pathway is associated with cell proliferation, migration, and invasion in GBM cells [5]. Additionally, the deregulation of genes in the G1/S cell cycle checkpoint was shown to be related to the development and proliferation of GBM cancer [6]. Previous studies reported, *VEGF* as one of the pro-angiogenesis factors secreted from glioma cells in the tumor microenvironment. *VEGF* also acts as a growth factor and its overexpression is correlated with poor prognosis and survival in GBM patients. VEGF receptor 2 (*VEGFR2*) as the primary effector of VEGF-A signaling, is expressed by endothelial and tumor cells to stimulate effects of VEGF signaling. Autocrine *VEGFR2* activation also promotes proliferation and self-renewal of glioblastoma stem-like cells [7, 8]. *PTEN* act as a suppressor of tumor progression and is a regulator of PI3K-mediated cell signaling pathways. It has been shown that in GBM, down-regulation of *PTEN* gene correlates with overexpression of the *AKT* proto-oncogene, which is located downstream of *PI3K*. Overexpression of *PTEN* was shown to induce cell cycle arrest in the G1 phase by reduction of nuclear localization of cyclin D1 [9]. Another important factor in controlling cell cycle progression is *p21*, a cyclin-dependent kinase (CDK) inhibitor. It has been reported that *p21* mediates a tumor suppressor function in the brain, through *p53* activity. Overexpression of *p21* was shown to induce tumor growth arrest through inhibition of cyclin-kinase complex, transcription factors, and coactivators [10]. Matrix-metallo proteinases (MMPs), play a crucial role in EMT and regulating the events that result in metastasis. Some MMP substrates cleavage products also have roles in cell growth, differentiation, apoptosis, migration, and angiogenesis regulation [11]. Hence, understanding the molecular mechanism, the function of genes, and the identification of biological biomarkers is important for prevention, further clinical treatment, and patient outcome improvement.

C-type lectins (CTLs) are superfamily proteins, some of which are secreted and others are transmembrane proteins. Some of the members of this superfamily have Ca^2+^ or glycan domain bands [12]. Interactions between tumor-specific glycans, lectins [13], calcium, and Ca^2+^ binding sites play key roles in diverse biological processes like cell adhesion, proliferation, metastasis, and invasion. Additionally, the role of Ca^2+^ signaling has been documented in different malignancies such as glioma [14], colorectal cancer [15], and melanoma [16]. CTLs can regulate many essential processes such as angiogenesis, metastasis, development, inflammation, immunological responses, and cell growth or cell death in cancers [17]. Previous studies have shown that *CLEC18B* [18], *CLEC12A* [19], and *CLEC11A* [20] are associated with the development of various cancers. In contrast, the number of CTLs members namely *CLEC3B* [21], *CLEC4G*/*M* [22], and *CLEC4E* [23] are decreased in cancer cells, which have the potential to be suppressor genes in different cancers.

*CLEC19A* (C-Type Lectin Domain Containing 19A) is a member of the C-type lectin family. *CLEC19A* is located on 16p12.3 and its gene products were detected as 3 protein-coding variants (Fig. S1A). The main isoform (NM_001256720) consists of 5 exons and the length of this polypeptide is 186 amino acids (Fig. S1B). Based on the human protein atlas database (https://www.proteinatlas.org) and NCBI [24] the *CLEC19A* mRNA is abundantly expressed in brain tissue compared to other human tissues (Fig. S1C). Our TMHMM and PSIPRED *in-silico* data, confocal microscopy imaging, and western blot analysis suggest that CLEC19A could be a secreted protein.

In this study, we analyzed the expression of *CLEC19A* from TCGA data of patients with LGGs (low-grade gliomas) and GBMs. We found that *CLEC19A* was downregulated in LGG and GBM tissues compared to normal brain tissues. Considering that the *CLEC19A* gene is expressed in the human brain and that its expression is likely decreased in the brain of glioma patients, we sought to study the *CLEC19A* gene in GBM. To this end, the expression level of *CLEC19A* was evaluated in glioma patients and glioma cell lines. We demonstrated that cell proliferation and migration were significantly decreased by overexpression of *CLEC19A* in U87 and C6 glioma cell lines. Also, the overexpression of *CLEC19A* was shown to be associated with the promotion of apoptosis and arrest of the cell cycle in the sub-G1 phase in the glioma cell lines. Our in vivo data revealed that the overexpression of *CLEC19A* significantly decreases brain tumor volume size and suppresses tumor growth in a glioma rat model.

## Materials and methods

### *In-silico* analysis for processing the RNAseq data and protein structure prediction

The transcriptome expression, along with clinical data of TCGA-GBM (https://www.linkedomics.org/data_download/TCGA-GBM/) and TCGA-LGG (https://www.linkedomics.org/data_download/TCGA-LGG/) datasets, were retrieved by the TCGA biolinks [25–27] package in R. Data on RNAseq and RSEM analyses of 166 GBM and 530 LGG, and 5 adjacent tumor samples were retrieved. The Shapiro–Wilk normality test was performed on each biological group, and the non-parametric Mann–Whitney U-test was chosen as an eligible test. This was followed by an evaluation of changes in a pair of groups for the *CLEC19A* expression. Further, the ggplot2 [28] and ggpubr [29] packages were utilized to visualize the data.

The sequence and orientation of the *CLEC19A* gene were surveyed using Entrez [30]. The *CLEC19A* mRNA expression level in brain tissues was retrieved from the HPA data in Human Protein Atlas database. ExPASy’s ProtParam tool [31] was employed to obtain and analyze physiochemical parameters. Moreover, secondary structural features of the protein were assessed via PSIPRED (PSI-blast-based secondary structure PREDiction), and the InterPro database was also used for Ca^2+^ and glycan-binding site prediction [32–34]. To test for the presence of transmembrane segments in CLEC19A, DeepTMHMM [35] and TOPCONS [36] online tools have been used. As the CLEC19A crystal structure is not available in the protein data bank, the CLEC19A protein reference sequence (NP_001243649.1) was used for three-dimensional structure prediction using 2 prediction software including UCSF ALPHAFOLD2 colab protein structure database [37] and Robetta online protein structure prediction server (https://robetta.bakerlab.org). Moreover, these servers were also used for 3D structure prediction of the CLEC19A/GFP fusion protein. To determine the quality of the predicted models, two validation servers including PROCHECK [38] and ERRAT [39] were used. The best-generated models based on the highest PROCHECK and ERRAT scores and also consideration of the domain structure of models were selected for additional evaluations. The predicted 3D models were then visualized using the UCSF Chimera program [40].

### Glioma tissue samples

Glioma samples were taken from the Imam Khomeini Educational and Medical Hospital, Sari, Iran by a neurosurgeon. Histological diagnosis and grading of tumors were confirmed by a pathologist in accordance with the WHO criteria [41]. Eight tumor samples were classified as LGG (grade II astrocytoma), and seven tumors were typed as GBM (grade IV glioblastoma). Four normal tissues were acquired from healthy brain tissues adjacent to the tumors. The samples were immediately frozen in liquid nitrogen and stored at − 80 °C until RNA isolation. This study was approved by the Ethics Committee of Tarbiat Modares University (No. IR.MODARES REC.1399.215) and was performed by the relevant guidelines and regulations (Declaration of Helsinki). Informed consent was obtained from all patients; patient information is shown in Table S1.

### Cell lines and culture conditions

A172 and U87 (Human glioblastoma cell lines), 1321N1 (Human brain astrocytoma), and C6 (Rat glioma cell) were cultured in Dulbecco's Modified Eagle's Medium/Nutrient Mixture F-12 (DMEM/F-12) (Gibco, USA). HEK293T (Human embryonic kidney) and NTERA-2 (Human testicular embryonal carcinoma) were cultured in DMEM-HG, supplemented with 1% antibiotics (10,000 U/mL of penicillin and 10, 000 μg/mL of streptomycin) (Gibco, USA), 10% fetal bovine serum (FBS) (Gibco, USA), and incubated at 37 °C with 5% CO2. The cell lines were obtained from the Pasteur Institute of Iran.

### RNA isolation, cDNA synthesis, and real-time PCR (qRT-PCR)

Total RNAs were extracted from the cell lines and frozen solid tissues using TRIzol™ reagent (Invitrogen, USA). The RNA concentration was determined by a Nanodrop ND-1000 spectrophotometer (Thermo Fisher Scientific, USA), and the sample’s 260/280 OD ratio was 2.0091 ± 0.001 (Mean ± SEM), demonstrating their high purity. The quality of RNA was verified by 1% agarose gel electrophoresis, indicating the 28S and 18S ribosomal RNA (rRNA). Subsequently, 1 μg of each RNA sample was treated with DNase I, RNase-free (Thermo Scientific, USA), and incubated at 37 °C for 30 min to remove any contaminating genomic DNA. Single-stranded cDNA was synthesized by the cDNA synthesis kit (Biotechrabbit, Germany) in a 20 μl reaction according to the manufacturer’s instructions, and stored at − 20 °C until use.

RT-PCR was performed using 1 μL of cDNA template with Phusion High-Fidelity PCR Master Mix (Thermo Fisher Scientific, USA) and 0.4 μM of each primer in a 20 μL PCR reaction according to the manufacturer's protocol. Real-time PCR was performed using RealQ plus 2X Master Mix Green, High ROX™ (Ampliqon, Denmark) in the Applied Biosystems StepOne Real-time PCR. *β-Actin* was used as an internal control. The relative expression levels and fold changes were calculated using the 2^−ΔCt^ and 2^−ΔΔCt^ formulas, respectively. Primers for genes studied herein were designed using Eurofins Genomics Primer Design Tool (https://eurofinsgenomics.eu) and verified using an IDT oligo analyzer (https://www.idtdna.com) and NCBI Primer-blast (https://www.ncbi.nlm.nih.gov). Primer and oligo sequences used in this study are listed in Table S2.

### Plasmid constructs and transient transfection

All of the fragments were amplified by Platinum® Taq DNA Polymerase High Fidelity (Invitrogen, USA), according to the manufacturer's instructions. For the cloning of the related fragments, the PCR products were purified using the BioBasic gel extraction kit (BioBasic, Canada), and cloned into pCDNA3.1( +) or pEGFP-N1 vectors. The full-length cDNA of the *CLEC19A* gene was amplified from the cDNA of the NTERA-2 (NT2) cell line and cloned into the pCDNA3.1( +) vector (Addgene) between the sites of EcoRI (Thermo Fisher Scientific, USA) and NotI (NEB, USA) (pCL19A) (Fig. S2A). To determine the localization of the *CLEC19A* gene, its ORF sequence without stop codon was fused to the EGFP sequence of a pEGFP-N1 vector (Addgene). Briefly, the ORF sequence was amplified from the cDNA of the NT2 cell line and inserted between the EcoRI (Thermo Fisher Scientific, USA) and SalI (NEB, USA) sites of the pEGFP-N1 vector in a frame with the EGFP sequence (pCL19A-ORF) (Fig. S2B). The sequences of all plasmid constructs were confirmed by colony PCR [42] and sanger sequencing [43].

The cells were transiently transfected with vectors used in this study using Lipofectamine ® 3000 (Thermo Fisher Scientific, USA). The cells were seeded into a plate 24 h before transfection. Next, the plasmid DNA-lipid complex was prepared according to the manufacturer's protocol and was added to cells.

### U87 and C6 stable cell lines generation

U87 and C6 cells were seeded into 6-well plates (1 × 10^5^/well) using DMEM/F-12 medium supplemented with 10% FBS and 1% antibiotics. After 24 h incubation, the cells were transfected with the pCL19A, pCDNA3.1 + , pCL19A-ORF, and pEGFP-N1 vectors (4 μg/well) using Lipofectamine3000 reagent (5 μL/well) (Thermo Fisher Scientific, USA). Two days after transfection, cells were treated with 2 mg/mL Neomycin (G418) (BioBasic, Canada) at 37 °C in a humidified atmosphere of 5% CO2 by killing the non-transfected cells for 14 days. The total RNAs were extracted according to the manufacturer's protocol, and the expression of the *CLEC19A* gene was assessed by qRT-PCR. Genomic DNA was obtained from cells using DNA Extraction Kit (QIAGEN, Germany) according to the manufacturer's instructions. The gDNA was amplified by PCR with the *CLEC19A* gene exon-exon junction primers, and the PCR products were analyzed in 1.5% of agarose gel.

### GFP detection by flow cytometry

For quantification of the fusion protein CLEC19A/GFP fluorescence signal, HEK293T cells (4 × 10^4^ cells/well) were seeded into a 24-well plate. After 24 h incubation, the cells were transfected with the pCL19A-ORF and pEGFP-N1 vectors (1 μg/well) using Lipofectamine3000 reagent (1 μL/well) (Thermo Fisher Scientific, USA). For negative control, HEK293T cells were used without any transfection. At 48 h after transfection, the cells were collected and washed with 1X PBS. The cells were assessed for the detection of fluorescent signals using the FACS Calibur Flow Cytometer (BD biosciences, USA), and the data were analyzed by FlowJo software (V10.8.1).

### Immunocytochemistry/Immunofluorescence (ICC/IF) assay

Stable U87 cells (2 × 10^4^ cells/well) were seeded into 4-well plates on coverslips. After 48 h, the cells were fixed in 4% Paraformaldehyde (Merck, Germany) for 10 min at room temperature. Next, the cells were washed three times with 1X PBS and permeabilized with 0.1% Triton X-100 (Merck, Germany) for 10 min at room temperature. After washing the cells in PBST (phosphate-buffered saline containing 0.1% Tween 20) (Bio basic, Canada) three times (5 min for each wash), a blocking solution containing 5% skim milk (Sigma, USA) was added to the cell and the cells were incubated for 2 h at 4 °C. Next, the cells were incubated with GFP antibody (B-2) Alexa Fluor® 488 (Santa Cruz, USA, sc-9996 AF488, 1:100) for 4 h at 4 °C in the dark. After three washes in PBST, the cells were treated with the β-Actin antibody (C4) Alexa Fluor® 647 (Santa Cruz, USA, sc-47778 AF647, 1:200) for 2 h at 4 °C in the dark, and the cells were washed in PBST three times. Finally, the cells were incubated for 2 min with 300 nM DAPI (Thermo Fisher Scientific, USA) as a nuclear stain. The cells were imaged using an inverted confocal microscope (LEICA MDi8) and LAS X software.

### Cell viability assay (MTT assay)

U87 and C6 cell lines (2000 cells/well) were seeded in 96-well plates in triplicate. After 24 h, the cells were transfected with pCL19A or pCDNA3.1( +) mock vectors (0.2 μg/well) using Lipofectamine3000 reagent (0.2 μL/well) (Thermo Fisher Scientific, USA). At 24 h, 48 h, 72 h, and 96 h post-transfection, 10 μL of 5 mg/mL Tetrazolium salt (Sigma, USA) were added to each well, followed by an additional incubation at 37 °C for 4 h. The cell supernatants were aspirated and discarded once the purple precipitate appeared in the wells. Next, 100 μL DMSO (Merk, USA) was added to each well to dissolve the formazan crystals followed by a measurement of optical density (OD) at 570 nm with an ELISA Microplate Reader (BioTek, USA).

### CFSE proliferation assay

Carboxyfluorescein succinimidyl ester (CFSE) working solution (1 μL of 5 mM CFSE dye in 1 mL PBS) (Thermo Fisher Scientific, USA) was added to the U87 and C6 cells (1 × 10^5^ cells per well of 12-well plates), and the cells were incubated for 20 min at 37 °C in the dark. After 24 h, the cells were transfected with pCL19A or pCDNA3.1( +) mock vectors (2 μg/well) using Lipofectamine3000 reagent (2 μL/well) (Thermo Fisher Scientific, USA), and incubated at 37 °C with 5% CO2 for three days. At 0 h, and 72 h the cells were assessed for proliferation using FACS Calibur Flow Cytometer (BD biosciences, USA), and the data were analyzed by FlowJo software.

### Colony-formation assay

DMEM/F-12 medium supplemented with 10% FBS, 1% antibiotics, and 0.5% agar was plated on the bottom of each well. Next, the U87 and C6 stable suspension cells in DMEM/F-12 medium supplemented with 10% FBS, 1% antibiotics, and 0.7% agarose were seeded (2500 cells/well in 6-well plates) on top of the agar layer. Plates were incubated at 37 °C with 5% CO2 for 2–3 weeks, and the medium was replaced every 2 days. Next, the cells were fixed with 4% paraformaldehyde (Merk, Germany) for 15 min, and stained using 0.05% crystal violet solution (Thermo Fisher Scientific, USA) for 60 min. Cell colonies were photographed and counted with ImageJ software (V1.53).

### Wound-healing assay

U87 and C6 (4 × 10^4^ /well) stable cells were seeded into 24-well plates. After 24 h, a straight linear wound was made across the confluent monolayer cell in each well using a sterile 200 μl pipette tip [44]. The cells were washed with PBS, cultured in DMEM/F12 supplemented with 10% FBS, and 1% antibiotics, and incubated at 37 °C with 5% CO2. The cell migration areas were scanned by measuring the distance between the two sides of the scratch after 120 h for U87 cells and 72 h for C6 cells using an inverted microscope (OLYMPUS IX53), and the images were analyzed with ImageJ software.

### Transwell assay

Six transwell filter chambers with 8 μm pores were used for the migration assay (SPL LIFE SCIENCES, Korea). 2 × 10^4^ U87 or C6 stable cells in 200 μl DMEM/F12 (Gibco, USA) without FBS were seeded on a polycarbonate membrane, and 600 μL DMEM/F12 with 10% FBS (Gibco, USA), and 1% antibiotics (10,000 U/mL of penicillin and 10,000 μg/mL of streptomycin) (Gibco, USA) was added to the lower compartment, and the chambers were incubated at 37 °C with 5% CO2 for 24 h. Next, the cells on the top surface of the chamber were removed with a cotton swab. The cells adhered to the lower surface were fixed with 4% paraformaldehyde (Merck, Germany) for 10 min at room temperature. After that, the cells were washed with 1X PBS three times and stained with 0.2% Crystal Violet solution (Thermo Fisher Scientific, USA) for 10 min at room temperature. Next, the cells were washed with DH2O three times to remove the Crystal Violet solution excess and were air dried at room temperature. The count of cells that had migrated through the pores was photographed with an inverted microscope (OLYMPUS IX53) in at least five areas, and the images were analyzed with ImageJ software.

### Cell cycle analysis

U87 and C6 cells (4 × 10^4^ cells/well) were cultured in 24-well plates in triplicates. After 24 h, the cells were transfected with pCL19A or pCDNA3.1( +) mock vectors (1 μg/well) using Lipofectamine3000 reagent (1 μL/well) (Thermo Fisher Scientific, USA). At 72 h, 96 h, and 120 h post-transfection, cells were harvested and fixed in 70% cold ethanol at 4 °C for 24 h. The fixed cells were stained with 500 μL PBS containing 50 μg/mL propidium iodide (Sigma, USA), 100 μg/mL RNase A (BioBasic, Canada), and 0.1% Triton X-100 (Sigma, USA), and incubated for 30 min at room temperature in the dark. All samples were assessed for the cellular DNA content by a FACS Calibur Flow Cytometer (BD biosciences, USA), and the data were analyzed by FlowJo software.

### Cell apoptosis assay

U87 and C6 cell apoptosis was assessed using Annexin V-PE/7AAD staining kit according to the manufacturer’s instruction (Roche, Germany). Briefly, the cells (4 × 10^4^ cells/well) were cultured in 24-well plates in triplicates and transfected as explained above. At 72 h, 96 h, and 120 h after transfection, the cells were washed with cold PBS and re-suspended in 1X binding buffer (10 mM HEPES/NaOH, PH 7.4, 140 mM NaCl, 2.5 mM CaCl_2_), followed by staining with Annexin V-PE/7AAD in the dark for 15 min at room temperature. The cells were evaluated for apoptosis using FACS Calibur Flow Cytometer (BD biosciences, USA), and the data were analyzed by FlowJo software.

### Protein extraction and western blotting

Stable U87 cells transfected by pCL19A and pCDNA3.1( +) vectors were lysed using Ripa buffer (BioBasic, Canada) on ice for 30 min according to the manufacturer’s protocol. The lysates were centrifuged at 13,000 rpm for 30 min, and the supernatant was collected and stored at -80 °C. The protein concentration was measured by the Bradford assay. To prepare the Bradford reagent, coomassie blue G-250 (Merck, Germany) was completely dissolved in methanol (Merck, Germany) and 85% phosphoric acid (H3PO4) (Merck, Germany). Next, the acid solution mixture was slowly added to H2O, and the solution was filtered with Whatman paper No1 (Merck, Germany) [45]. To evaluate the concentration of standard protein (BSA) and protein samples, the Bradford reagent was added to each sample, and absorbance was measured at 630 nm using a spectrophotometer. Total protein was subjected to SDS-PAGE using a 10% polyacrylamide gel and transferred to a PVDF membrane (Thermo Fisher Scientific, USA). Next, membrane blocking was performed at 4 °C for 2 h using 5% skim milk (Sigma, USA) diluted in PBST (Bio basic, Canada). Subsequently, the membrane was incubated with the primary antibodies to GFP (Santa Cruz, USA, sc-9996, 1:300), MMP2 (Santa Cruz, USA, sc-10736, 1:300), RECK (Santa Cruz, USA, sc-373929, 1:300), BAX (Santa Cruz, USA, sc-7480, 1:300), BCL2 (Santa Cruz, USA, sc-492, 1:300), IκB-α (Santa Cruz, USA, sc-1643, 1:300), TIMP3 (Santa Cruz, USA, sc-373839, 1:300), PI3K (Santa Cruz, USA, sc-67306, 1:300), and β-Actin (Santa Cruz, USA, sc-47778, 1:300) overnight at 4 °C. The anti-mouse IgG-Horse Radish Peroxidase (HRP) (Santa Cruz, USA, sc-516102, 1:1000) or anti-rabbit IgG-HRP (Santa Cruz, USA, sc-2357, 1:1000) secondary antibodies were diluted according to the manufacturer’s instructions and was incubated with the membrane for 1 h after three times washes with PBST. Signals were detected with an ECL detection reagent (Thermo Fisher Scientific, USA). β-Actin protein was used for normalization.

### In vivo tumor xenograft model (surgery, implantation, and MRI)

To investigate a potential therapeutic effect of overexpression of the *CLEC19A* gene in vivo, the glioblastoma rat models were generated by stable C6 cell lines with pCL19A (overexpression construct), pCDNA3.1 + (mock) and C6 cell lines (untreated). Eighteen male Wistar rats (untreated group = 6n, Mock group = 6n, and PCL19A group = 6n) in the weight range of 200–250 gr, were purchased from Pasteur Institute in Iran. The Animal Experimental Ethics Committee of the Tarbiat Modares University approved the animal studies (No. IR.MODARES REC.1399.215) and we did our best to alleviate animal suffering.

The rats were kept under 12 h of darkness and light conditions with free access to food and water in the animal house of Tarbiat Modares University. For this experiment, rats were anesthetized with ketamine 100 mg/kg and xylazine 10 mg/kg and were restrained in the stereotaxic apparatus. A longitudinal incision was made on the animal's head. The skin and underlying tissues were removed and the bregma and lambda points were exposed. Next, 1 × 10^6^ C6 cells from each group (untreated, Mock, and overexpression construct) were injected into the Caudate Putamen striatum (AP): -2 mm, (ML): 2 mm, and (DV): -4 mm [46] according to the Paxinus Watson atlas, by infusion pump (Fig. S3). Finally, animals were transferred to cages for recovery.

Twenty days after surgery, the Magnetic Resonance Imaging (MRI) system (T2 method) (3 T MAGETOM Prisma, Siemens, Germany) was used to assess tumor growth in different experimental groups. MRI images were taken from an average of 8 transverse slices with a thickness of 0.8 mm. After determining the tumor margin in each section, the tumor dimensions were calculated automatically by ITK_SNAP software.

### Statistical analyses

All experiments were performed in triplicates independently, and the results were reported as the means ± standard error means (SEM). *P-*values < 0.05 were considered statistically significant. The t-test (when comparing two groups) or One-Way Analysis of Variance (ANOVA) (when comparing three groups) was performed using GraphPad Prism 9.4 software to determine significant differences in measured variables between groups. RNAseq data analysis was performed by Mann–Whitney U-test.

## Results

### *In-silico* protein modeling of CLEC19A suggested that CLEC19A is a secreted protein

**Fig. 1:**
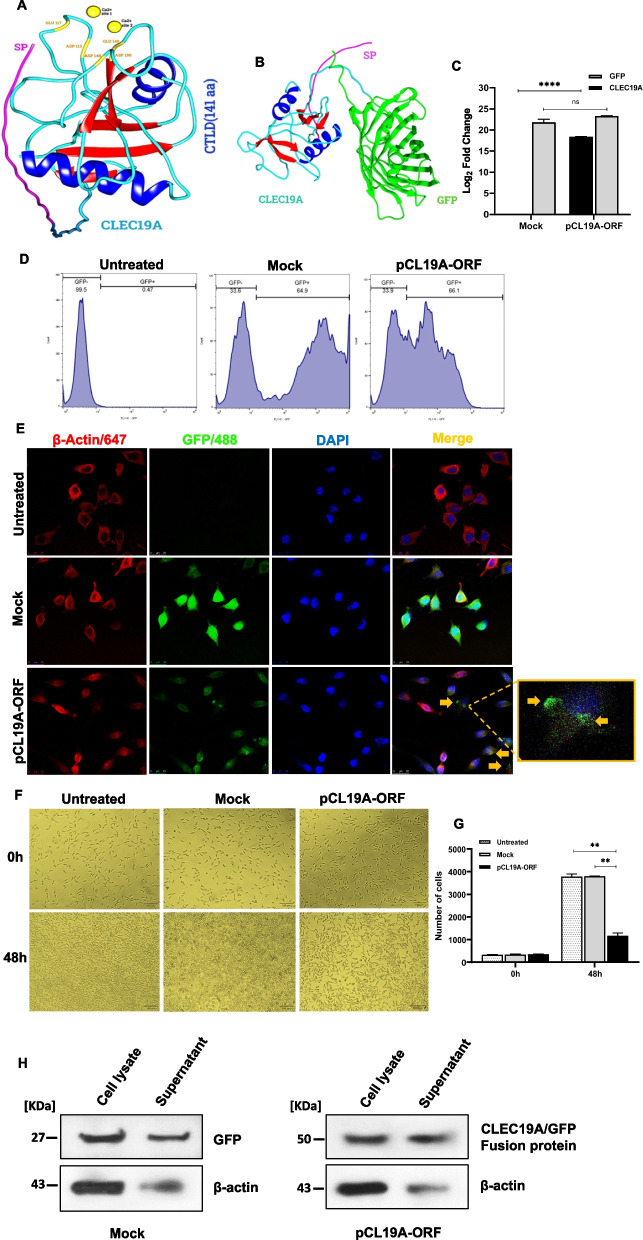
Analysis of CLEC19A protein models and localized protein in U87 cells that indicated CLEC19A is a secreted protein and decreased cell proliferation. **A** 3D schematic illustration of the CLEC19A protein structure with the Chimera program. CLEC19A consists of a C-type lectin domain (CTLD) and signal peptide (SP). Two calcium binding sites are marked in yellow color in the figure. **B** 3D schematic illustration of the CLEC19A/GFP fusion protein structure with the Chimera program. As shown in figure B, the fusion of GFP does not affect the CLEC19A protein structure. **C** Bar plot analysis shows the CLEC19A and GFP expression levels in U87 cells, which indicated transcription of the CLEC19A/GFP full-length. **D** GFP fluorescence intensity in HEK293T cells transfected by pEGFP-N1 mock and pCL19A-ORF vectors, and untreated cells using flow cytometry. fluorescence intensity indicated the correct folding of GFP and CLEC19A proteins in the fusion protein. **E** ICC/IF staining. The confocal microscopy imaging showed that CLEC19A/GFP fusion proteins were on the cell membrane and extracellular regions, while the GFP proteins were located in the cytoplasm. **F** The U87 light microscope images. The culture medium from U87 untreated cells, mock cells, and pCL19A-ORF were collected after 48 h and added to U87 cells. **G** The results show the cell proliferation in the supernatant of the cells in which transfected by pCL19A-ORF was significantly lower compared to the untreated and mock (Cells were photographed and counted with ImageJ software). **H** After CLEC19A overexpression Western blot analysis of cell lysate and supernatant, showed that CLEC19A is probably a secreted protein. (β-Actin was used as a control [47]). Columns, mean of three different experiments. Statistical analysis was conducted using a one-way ANOVA or student t-test and means ± SEM was shown. ns = not significant, **p < 0.01, ****p < 0.0001

Given that little information is available about CLEC19A protein, we studied the structure and location of CLEC19A protein with *in-silico* and in vitro experiments. Analysis of physicochemical parameters of CLEC19A protein sequence (NP_001243649.1) from ExPASy’s ProtParam has shown that this protein has the isoelectric point 4.9, aliphatic index 67.15, and has more negatively charged residues (24 aa) than positively charged amino acids (13 aa). Among all residues, the number and the percentage of Ser (9.7%) are the highest. The results of the DeepTMHMM and TOPCONS servers have shown that the CLEC19A protein has an N-terminal signal peptide and no transmembrane domain, which suggests that this protein is likely a secreted protein (Fig. S4A, B). Application of the InterPro database revealed that the CLEC19A protein, similar to several other proteins in this superfamily, is likely to have the propensity of binding to carbohydrates and calcium ions. Template-free modeling (de novo or ab initio) approach from Robetta and UCSF ALPHAFOLD2 colab servers was used for the prediction of 3D structures of CLEC19A protein and CLEC19A/GFP fusion protein (Used for CLEC19A localization). The schematic 3D illustrations of the protein structures with the Chimera 1.16 program are shown in Fig. 1 (A, B). As shown in Table S3, the accuracy of models is assessed by ERRAT and PROCHECK. ERRAT server recognizes incorrect regions of protein structures in random dispersions of atoms, which can be differentiated from correct distributions. Predicted models from Robetta and ALPHAFOLD2 showed scores in the range between 53.25–81.69% for the CLEC19A protein and 73.91- 81.79% for the CLEC19A/GFP fusion protein. PROCHECK server evaluates the stereochemical quality of protein structures residue-by-residue and the overall structural geometry of proteins. It presents results in the range from 66–86.9% for the CLEC19A and 78–86.9% for CLEC19A/GFP fusion protein. Validation scores and domain structure consideration suggest that model 4 from Robetta is the best model among the CLEC19A protein structures, and model 2 from ALPHAFOLD2 is the best prediction for CLEC19A/GFP fusion protein (Table S3). Structural alignment performed between CLEC19A and CLEC19A/GFP best prediction models using the Chimera structure comparison tool revealed that the fusion of GFP does not affect the CLEC19A protein structure. Molecular structure analysis of CLEC19A demonstrated that it contains a signal peptide at its N-terminus from amino acid residue 1 to amino acid 19, with a C-type lectin domain from amino acid 40 to 180 and 2 disulfide bridges (Fig. S4C).

### Imaging and immune blot analyses showed that CLEC19A is a secreted protein and its overexpression is associated with a decreased cell proliferation

Guided by the *in-silico* prediction analysis results, we ascertained the cellular localization of CLEC19A protein in U87 cells. The entire coding region without termination codon was cloned into the N-terminal of the pEGFP-N1 vector. At first, plasmids containing the verified sequence (pCL19A-ORF) were transfected into U87 cells, and the expression levels of CLEC19A and GFP were detected by qRT-PCR. The GFP fluorescence intensity of fusion protein was assessed using flow cytometry in HEK293T cells. The ICC/IF and Western blot analyses were also used for fusion protein localization in U87 stable cells. As shown in Fig. 1C, the CLEC19A and GFP expression levels were statistically significantly increased (*p* < 0.0001) in U87 cells transfected by pCL19A-ORF compared to mock (pEGFP-N1) cells, which indicated transcription of the CLEC19A/GFP full-length. Flow cytometry analysis detected the GFP fluorescence intensity in cells transfected by mock and pCL19A-ORF vectors. Although lower fluorescence intensity was observed in the HEK293T cells transfected by pCL19A-ORF compared to the mock, the result indicated the correct folding of GFP and CLEC19A proteins in the fusion protein (fusion protein structure and folding predicted in ALPHAFOLD2 software). The GFP fluorescence signal was not observed in untreated cells (Fig. 1D). For ICC/IF staining β-actin antibody/Alexa Fluor 647, GFP antibody/Alexa Fluor 488, and DAPI were used to visualize cytoplasmic microfilament, GFP or fusion protein, and nucleus, respectively. The confocal microscopy imaging showed that CLEC19A/GFP fusion proteins were majorly on the cell membrane and in extracellular matrix (ECM), while the GFP proteins in the mock-treated cells were located in the whole cytoplasm (Fig. 1E). To confirm confocal microscopy data, the supernatant after 48 h from U87 untreated cells, cells transfected by mock vectors, and cells transfected by pCL19A-ORF vectors collected were added to the U87 cells. After 48 h, the cells were observed under a light microscope. Treatment of the U87 cells with the supernatant of pCL19A-ORF transfected cells statistically significantly decreased cell proliferation compared to untreated and mock (*p* < 0.01) (Fig. 1F, G). Further, Western blot analysis demonstrated that CLEC19A protein is present in supernatant in U87 cells transfected by pCL19A-ORF to a greater extent compared to the mock group (5.14-fold) (Fig. 1H).

All in all, these results indicate that CLEC19A is plausibly a secreted protein and it can decrease cell proliferation.

### Differential expression of* CLEC19A* gene in human glioma tissues and glioma cell lines

**Fig. 2:**
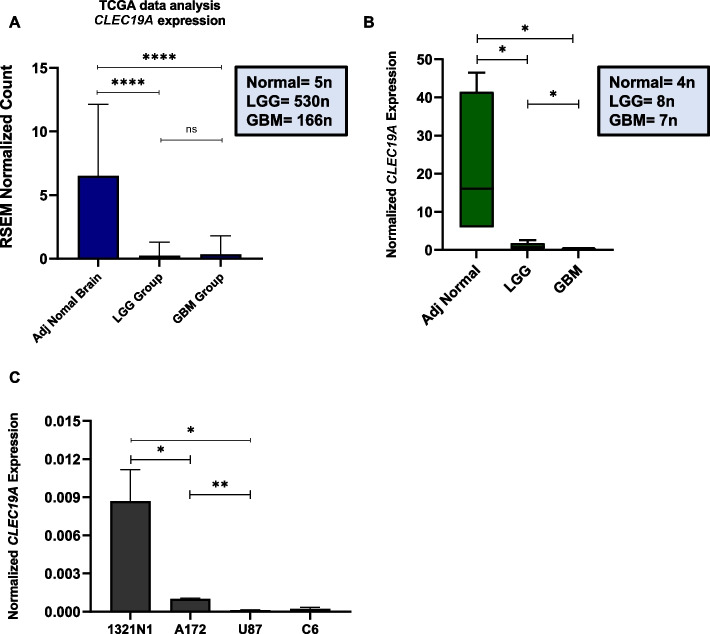
Expression of *CLEC19A* gene in human cancerous and normal brain tissues and brain-derived cell lines. **A** The *CLEC19A* expression in LGG and GBM samples compared to normal samples from TCGA data (total samples = 701). The expression level of *CLEC19A* in adjacent normal samples is significantly higher than in LGG and GBM samples. **B** The *CLEC19A* expression in human glioma tissues compared to adjacent normal samples was detected by qRT-PCR (total samples = 19). *CLEC19A* expression level is significantly lower in LGG and GBM patients compared to normal samples. **C** The transcription level of the *CLEC19A* gene in the 1321N1 low-grade astrocytoma cell line and A172, U87, and C6 GBM cell lines. The *CLEC19A* expression level decreased in GBM cell lines compared to the low-grade astrocytoma cell line (1321N1). Columns, mean of three different experiments. Statistical analysis was conducted using a one-way ANOVA or student t-test and means ± SEM was shown. ns = not significant, *p < 0.05, **p < 0.01, ****p < 0.0001

Based on NCBI and the Human protein atlas data *CLEC19A* has the highest expression in brain tissue compared to other human tissues, GBM and LGG RNAseq data were obtained from the TCGA database to evaluate *CLEC19A* expression in glioma and normal tissues. Analyzing samples from the TCGA dataset consisting of adjacent tumor samples, LGG, and GBM tissues demonstrated that *CLEC19A* gene expression statistically significantly decreased in LGG and GBM tissues compared to adjacent normal brain tissues (*p* < 0.0001) (Fig. 2A). To ascertain this, we next investigated the expression of the *CLEC19A* gene in samples from LGG (*n* = 8), GBM (*n* = 7), and adjacent tumor samples (*n* = 4) using qRT-PCR. The qRT-PCR results revealed that LGG and GBM samples expressed a statistically significantly lower level of *CLEC19A* gene compared to normal brain tissues (*p* < 0.05), consistent with bioinformatic data (Fig. 2B). To verify further the down-regulated *CLEC19A* expression in glioma, the level of *CLEC19A* was assessed in 1321N1, A172, U87, and C6 cell lines. The results corroborated that *CLEC19A* expression is statistically significantly reduced in aggressive glioma cell lines including A172, U87, and C6 relative to 1321N1 low-grade astrocytoma cells (*p* < 0.05) (Fig. 2C). These results show that *CLEC19A* expression decreases in glioma tumors compared to normal brain tissues and its expression is significantly lower in aggressive glioma cell lines compared to1321N1 low-grade astrocytoma cell line.

### *CLEC19A* overexpression decreased the cell viability and proliferation in U87 and C6 cell lines

**Fig. 3:**
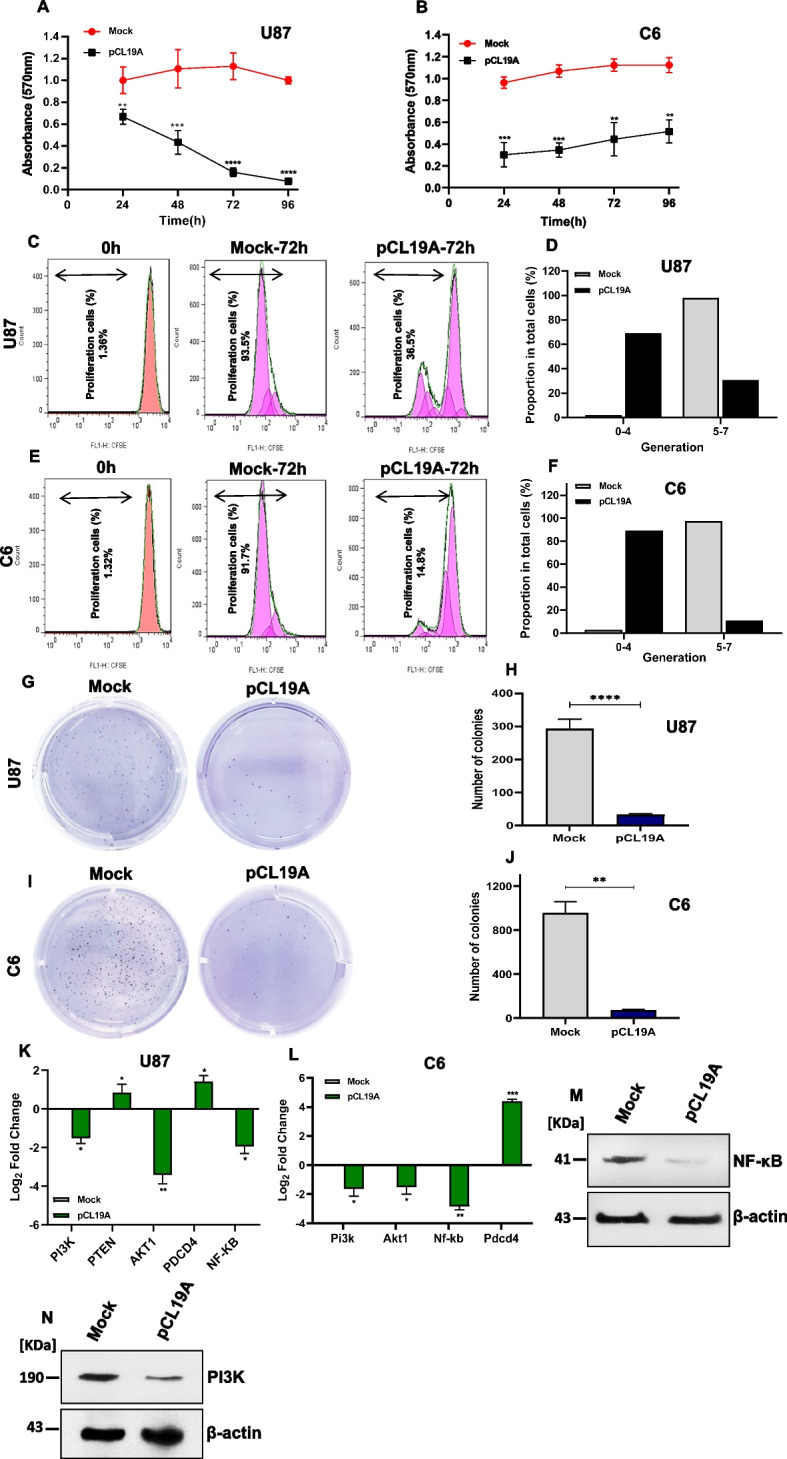
*CLEC19A* overexpression affects cell viability and proliferation in glioma cells. **A**, **B** MTT assays in U87 and C6 cells after transfection with pCL19A and pCDNA3.1( +) mock vectors. The cell viability was significantly decreased in all time points tested after overexpression of *CLEC19A* in U87 and C6 cells. **C**, **D** CFSE assay was performed to determine the proliferation rate following transfection of pCL19A and mock vectors in U87 cell line. The overexpression of the *CLEC19A* gene arrested cell proliferation after 72 h. **E**, **F** CFSE assay at 0 h and 72 h in C6 cells. Overexpression of *CLEC19A* inhibited cell proliferation in the C6 cell line. **G**, **H** Colony formation assay of U87 stable cells transfected by pCL19A and mock vector after 14 days. The number of colonies was significantly decreased in U87 cells after overexpression of *CLEC19A*. **I**, **J** Colony formation assay of C6 stable cells transfected by pCL19A and mock vector. The colony count was considerably lower in C6 plates transfected with pC19A compared to the mock group. **K**, **L** *PI3K*, *PTEN*, *AKT1*, *NF-κB*, and *PDCD4* mRNA Expression levels in U87 and C6 stable cells. The analyses indicated that *CLEC19A* overexpression caused *PI3K*, *AKT1* and *NF-κB* mRNA level reduction. The *PDCD4* gene expression was significantly up-regulated in U87 and C6 cells transfected by pCL19A compared to the mock. qRT-PCR analysis showed up-regulation of the *PTEN* mRNA level in U87 stable cells after *CLEC19A* overexpression. **M**, **N** Western blot analysis of NF-κB and PI3K protein levels in U87 stable cells transfected by pCL19A and mock vector. β-Actin was used as endogenous control. The NFκB and PI3K protein levels were decreased in U87 stable cells transfected by pCL19A compared to the mock. Columns and points, mean of three different experiments. Statistical analysis was conducted using a one-way ANOVA or student t-test and means ± SEM was shown. *p < 0.05, **p < 0.01, ***p < 0.001, ****p < 0.0001

To investigate the effect of *CLEC19A* overexpression on the survival of the glioma cell lines, the MTT assay was performed at 24 h, 48 h, 72 h, and 96 h. The results demonstrated that the viability of the U87 and C6 cells statistically significantly decreased in transfected cells with pCL19A compared to pCDNA3.1( +) mock-transfected cells in all time points tested (*p* < 0.05) (Fig. 3A, B). To explore the functional role of *CLEC19A* in the inhibition of cell proliferation, U87 and C6 cells were labeled with carboxyfluorescein succinimidyl ester (CFSE), and cells were transfected with pCL19A or mock vectors. After 72 h, flow cytometry data showed the ability to inhibit the cell proliferation of glioma cell lines transfected by pCL19A (Fig. 3C, E), as revealed by CFSE staining.72 h after the transfection of cells with pCL19A, the overexpression of the *CLEC19A* gene caused most of the cells to stop at 0–4 generation, and the cells transfected with mock were placed at 5–7 generation, indicating the arrest of cell proliferation in cells that were transfected with pCL19A (Fig. 3D, F) in U87 and C6 cell lines. Additionally, overexpression of *CLEC19A* remarkably reduced the colony counts in U87 and C6 stable cells compared to mock-transfected stable cells (Fig. 3G, I). The number of colonies was statistically significantly decreased following the overexpression of *CLEC19A* in U87 and C6 cells, compared to mock cells (*p* < 0.01) (Fig. 3H, J). The qRT-PCR analysis of *PI3K*, *PTEN*, *AKT1*, *NF-κB*, and *PDCD4* gene expression in U87 and C6 cells indicated that *CLEC19A* overexpression was associated with a significant reduction in *PI3K*, *AKT1* and *NFκB* mRNA levels. The *PDCD4* gene expression was statistically significantly up-regulated in U87 and C6 stable cells transfected by pCL19A compared to mock cells (*p* < 0.05). The expression of the *PTEN* gene was also significantly increased after *CLEC19A* overexpression in U87 stable cell lines (*p* < 0.05) (Fig. 3K, L). To support the idea that *CLEC19A* overexpression can decrease proliferation, the protein level of NF-κB and PI3K was measured by Western blot analysis in U87 stable cell lines (transfected by pCL19A and mock). The results showed that the protein level of NF-κB and PI3K was decreased in U87 cells transfected by pCL19A compared to mock-transfected cells (0.34-and 0.53- fold reduction respectively) (Fig. 3M, N). Together, these results confirmed that overexpression of the *CLEC19A* gene is associated with a reduction in cell viability and proliferation.

### Overexpression of *CLEC19A* inhibited glioblastoma cell migration

**Fig. 4:**
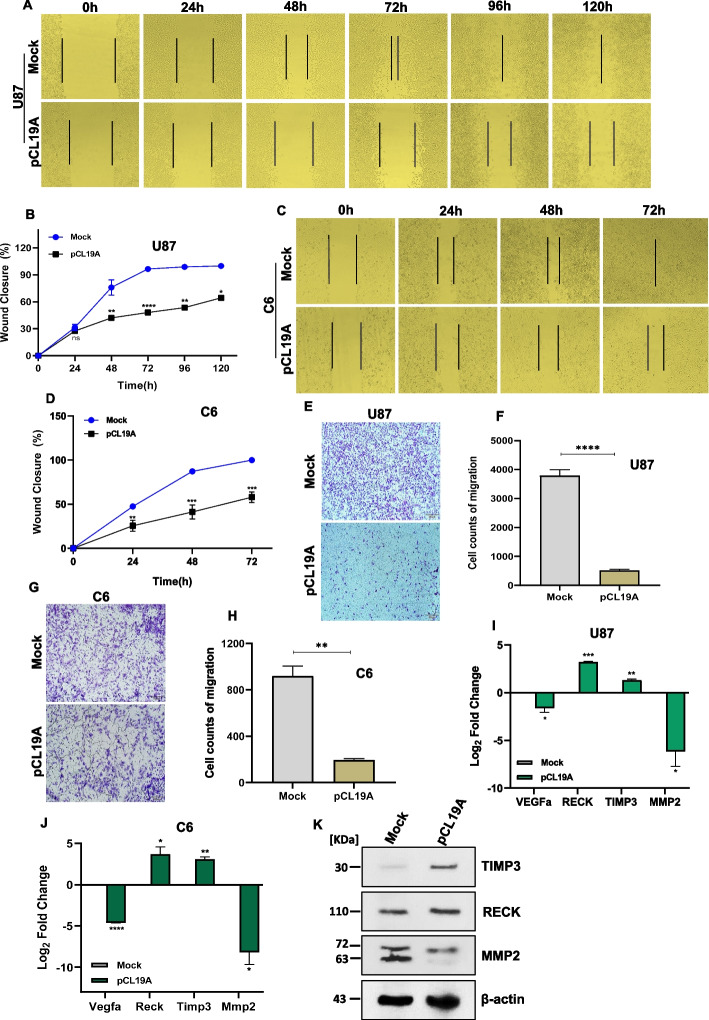
Effects of *CLEC19A* overexpression on cell migration. **A**, **B** In vitro wound healing analysis of U87 stable cells transfected with pCL19A and mock vectors at 0 h, 24 h, 48 h, 96, and 120 h post-scratching. *CLEC19A* overexpression in U87 cells significantly decreased cell migration in all time points tested. **C**, **D** wound healing assay of C6 stable cells following transfection with pCL19A and mock vectors at 0 h, 24 h, 48 h and 72 h. The overexpression of *CLEC19A* could decline the cell migration rate in C6 cells. **E**, **F** Transwell migration assay was administrated 24 h after seeded U87 stable cells on chambers. The number of migrated cells was significantly decreased in the U87 cell line after 24 h. **G**, **H** Show transwell migration assay in C6 stable cells after 24 h post-seeding. The migrated cell counts indicate that migration ability was significantly reduced in C6 cells transfected with pCL19A compared to mock cells. **I**, **J** *VEGFα*, *RECK*, *TIMP3*, and *MMP2* mRNA levels in U87 and C6 cells. The qRT-PCR results suggest that overexpression of *CLEC19A* significantly decreases the migration potential of glioma cancer cells. **K** Western blot analysis of TIMP3, RECK, and MMP2 protein levels in U87 stable cells transfected by pCL19A and mock vector. β-Actin was used as endogenous control. A decrease in the MMP2 protein level and an increase in the TIPM3 and RECK protein levels indicated that overexpression of *CLEC19A* reduced the ability of U87 cancer cell migration. Columns and points, mean of three different experiments. Statistical analysis was conducted using a one-way ANOVA or student t-test and means ± SEM were shown. ns = not significant, *p < 0.05, **p < 0.01, ***p < 0.001, ****p < 0.0001

To evaluate the effect of *CLEC19A* overexpression on glioma cell migration in vitro, we explored its impact on the migration of U87 and C6 cells using a wound healing assay, transwell test, and Western blot analysis. Firstly, a scratch wound healing assay was applied at different time points in U87 and C6 stable cell lines. Results showed that *CLEC19A* overexpression in U87 and C6 cells decrease migration toward the scratch wound (Fig. 4A, C) and statistically significantly reduced the number of migrative cells in both cell lines in all time points tested (*p* < 0.05) (Fig. 4B, D). Secondly, a Transwell assay was used to investigate the role of *CLEC19A* overexpression on U87 and C6 stable cell migration. *CLEC19A* overexpression was associated with a decreased number of migrated cells compared to pCDNA3.1( +) mock-transfected cells in U87 and C6 cell lines (Fig. 4E, G). The number of migrated cells statistically significantly reduced after 24 h in U87 and C6 stable cells (*p* < 0.01) (Fig. 4F, H). Moreover, to examine the expression levels of *VEGF*α, *RECK*, *TIMP3*, and *MMP2* mRNA levels in U87 and C6 Stable cells, the related cDNA samples were amplified using qRT-PCR. Overexpression of *CLEC19A* in both U87 and C6 cells led to significantly a reduced expression of the *VEGFα* and *MMP2* genes and an increased expression of the *RECK* and *TIMP3* genes (*p* < 0.05) (Fig. 4I, J). Furthermore, to determine the *CLEC19A* effect on cell migration, the protein level of TIMP3, RECK, and MMP2 was assessed in U87 stable cells using Western blot. An increase in the TIPM3 and RECK protein levels (3.21- and 1.35-fold increase respectively) and a decrease in the MMP2 protein level (0.46-fold) indicated that overexpression of *CLEC19A* reduced the ability of U87 cancer cell migration (Fig. 4K). These results suggest that overexpression of *CLEC19A* significantly decreases the migration potential of glioblastoma cancer cells.

### Overexpression of *CLEC19A* arrested the cell cycle and promoted apoptosis in U87 and C6 cells

**Fig. 5:**
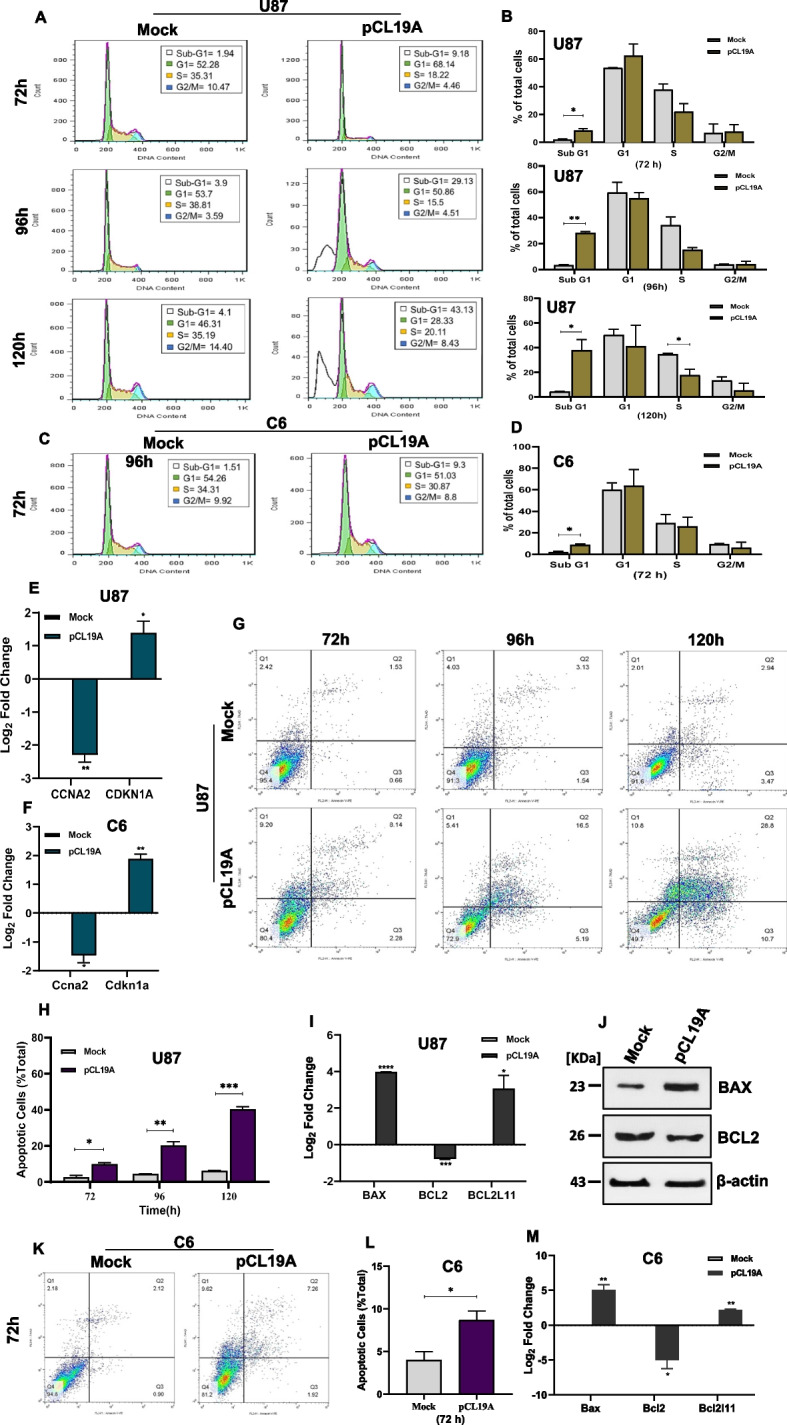
Effects of *CLEC19A* overexpression on cell cycle and apoptosis in U87 and C6 cells. **A** Shows PI staining flow cytometry analysis of U87 cells transfected with pCDNA3.1( +) mock vector or the vector which ensured *CLEC19A* overexpression in 72 h, 96 h, and 120 h. **B** Shows the percentage of the cells in each phase of the cell cycle, following the *CLEC19A* overexpression. Sub-G1 cell proportion has increased due to *CLEC19A* overexpression in the U87 cell line in all time points tested. **C** PI staining flow cytometry analysis of C6 cells transfected with mock or pCL19A vectors in 72 h. **D** The sub-G1 phase has risen in C6 cells transfected by pCL19A compared to the mock group. **E** Bar graphs represent the relative expression of *CCNA2* activity calculated from each group in U87 and C6 cell lines. The expression level of *CCNA2* was significantly decreased in both cell lines. **F** The *CLEC19A* overexpression caused a significant increase of *CDKN1A* expression at both cell line, suggesting that the overexpression of *CLEC19A* may regulate cell cycle in GBM cells. **G** Cell apoptosis assay was performed to determine the apoptosis rate following transfection of pCL19A and mock vectors in U87 cells. **H** Apoptotic cell percentage of total cells was increased at 72 h, 96 h, and 120 h in U87 cell line. **I** mRNA Expression level of *BAX*, *BCL2*, and *BCL2L11* in U87 cells after transfection with pCL19A and mock vectors. The results show the expression levels of *BAX* and *BCL2L11* were considerably higher in cells after transfection with pCL19A compared to the mock. Instead, the *BCL2* expression level was significantly reduced in cells transfected with pCL19A compared to mock-transfected cells. **J** Western blot analysis shows upregulation of BAX and downregulation of BCL2 by *CLEC19A* overexpression. **K** Cell apoptosis assay in C6 cells at 72 h after transfection by pCL19A and mock vectors. **L** Apoptotic cell percentage of total cells was increased at 72 h in C6 cell line after transfection by pCL19A compared to mock. **M** *Bax*, *Bcl2*, and *Bcl2l11* mRNA Expression levels in C6 cells after transfection. The *Bcl2* expression level was significantly lower in cells transfected with pCL19A compared to the mock, while *Bax* and *Bcl2l11* mRNA expression levels significantly increased in C6 cells transfected with pCL19A compared to the mock. Columns, mean of three different experiments. Means ± SEM was shown. Statistical analysis was conducted using student t-test or one-way ANOVA. *p < 0.05, **p < 0.01, ***p < 0.001, ****p < 0.0001

Next, we examined the potential impact of *CLEC19A* overexpression on the cell cycle and apoptosis of glioma cell lines. The inhibitory role of *CLEC19A* overexpression on the U87 and C6 cell cycle was investigated by cell cycle analysis at different time points after transfection (72 h, 96 h, and 120 h). Further, the *CCNA2* (*Cyclin A2*) and *CDKN1A* (*P21*) cell cycle genes expression level was evaluated in the U87 and C6 stable cell lines (Transfected by pCL19A and pCDNA3.1( +) mock). Cell cycle analysis illustrated that overexpression of *CLEC19A* compared to mock-transfected cells is associated with the arrest of the cell cycle in the sub-G1 phase (*p* < 0.05), with a concomitant decrease in the proportion of S phase in all time points tested in U87 cells. At 120 h, 44.13% of cells were arrested at the sub-G1 phase (Fig. 5A, B). In agreement with the U87 cell cycle results, *CLEC19A* overexpression in C6 cells was associated with a statistically significant increase in sub-G1 arrest at 72 h (*p* < 0.05) (Fig. 5C, D). Further, the effect of *CLEC19A* overexpression on the expression level of the cell cycle-associated gene, *CCNA2* gene expression was examined. The results showed that overexpression of *CLEC19A* statistically significantly decreased the mRNA expression level of *CCNA2* in U87 and C6 stable cells compared to mock-transfected stable cells (*p* < 0.01) (Fig. 5E). Evaluation of the expression level of *CDKN1A* in the CLEC19A overexpressed U87 and C6 stable cell lines revealed that the *CDKN1A* gene expression level is statistically significantly increased compared to the mock treated cell lines (*p* < 0.01) (Fig. 5F). Based on a significant increase in the sub-G1 phase, we employed the Annexin V-PE staining method to investigate the apoptosis rate in the U87 cells transfected by pCL19A and mock at 72 h, 96 h, and 120 h. Further, the level of apoptotic and anti-apoptotic genes and proteins was evaluated using a qRT-PCR and western blot analysis in the U87 stable cell lines (Transfected by pCL19A and pCDNA3.1( +) mock). For apoptosis analysis in the C6 cell line, the cells were transfected by pCL19A or mock vectors. In addition, the level of apoptotic and anti-apoptotic genes was determined using a qRT-PCR in C6 stable cells. Figure 5G and H show that the proportions of U87 apoptotic cells following the *CLEC19A* overexpression were statistically significantly increased, compared to those in the mock group in all time points tested (*p* < 0.05). Further, *CLEC19A* overexpression statistically significantly inhibited the protein and mRNA levels of anti-apoptotic BCL2 (*p* < 0.001) and statistically significantly increased the level of BAX protein and mRNA (*p* < 0.0001), as well as *BCL2L11* (*BIM*) mRNA level (*p* < 0.05) (Fig. 5I, J). Compared to the mock group, a significant increase in BAX/BCL2 ratio (3.46-fold) was observed in cells transfected by pCL19A. Similarly, the proportions of apoptotic cells in C6 cells transfected by pCL19A rose statistically significantly compared to mock (*p* < 0.05) (Fig. 5K, L). Analysis of the expression levels of *Bax*, *Bcl2*, and *Bcl2l11* in C6 stable cells revealed that the Bcl2 gene expression level is statistically significantly declined (*p* < 0.05). In contrast, *Bax* and *Bcl2l11* mRNA levels were statistically significantly increased (*p* < 0.01) (Fig. 5M). These results indicate that the overexpression of *CLEC19A* could arrest cell progression in the sub-G1 phase of the cell cycle and induce apoptosis in U87 and C6 glioma cell lines.

### *CLEC19A* gene overexpression suppressed brain tumor growth in a rat model of glioblastoma

**Fig. 6:**
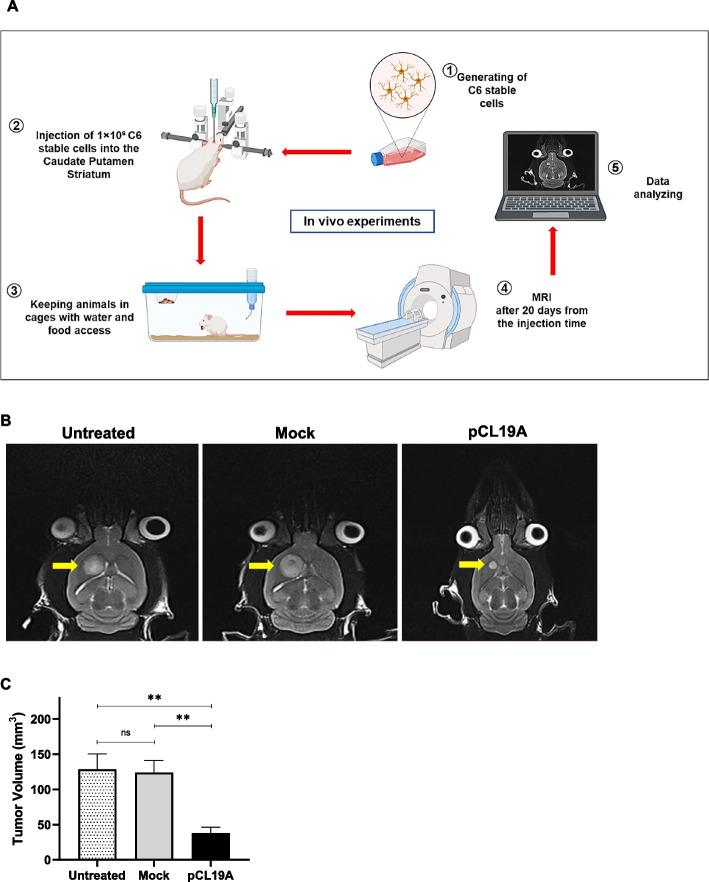
In vivo experiments. **A** Schematic image of in vivo experiment for delivering C6 stable cells in the brain of healthy rats and detecting tumor condition by MRI. (Parts of the figure are drawn by the BioRender site). **B** Images of glioma tumors in different groups (untreated group = 6n, Mock group = 6n, and PCL19A group = 6n). The MRI results revealed that overexpression of *CLEC19A* in rat brains could inhibit tumor growth. **C** Bar plot analysis shows that *CLEC19A* overexpression can reduce tumor size compared to untreated and mock groups. The volume size of the tumor was calculated by ITK_SNAP software. Statistical analysis was conducted using a one-way ANOVA or student t-test and means ± SEM was shown. ns = not significant, **p < 0.01

Our in vitro data showed that the overexpression of *CLEC19A* in C6 cells significantly decreased cell proliferation, viability, and migration. Besides, *CLEC19A* overexpression could arrest the cell cycle in the sub-G1 phase and induce apoptosis. To substantiate the in vitro results, in vivo experimentation was carried out in male Wistar rats. Brain tumor volume (mm3) was measured on day 20 of brain rats injected stereotaxically with 1 million ether C6 cells (untreated group), C6 stable cells transfected by pCDNA3.1( +) mock (mock group), or C6 stable cells transfected by pCL19A (*CLEC19A* overexpression group) (Fig. 6A). The MRI results revealed that overexpression of *CLEC19A* in rat brains could statistically significantly suppress tumor growth rate in comparison to untreated and mock groups (Fig. 6B, C; Fig S5). Collectively, these results indicate that *CLEC19A* overexpression can inhibit the tumorigenicity of C6 glioma cells in vivo, and significantly decreases brain tumor volume size in the glioma rat model.

## Discussion

Glioblastoma multiform (GBM) is regarded as an aggressive type of brain cancer [48] and is more common in adults [49, 50]. A better understanding of molecular mechanisms and the discovery of biomarkers for early diagnosis of GBM may help improve diagnosis and decrease the rate of recurrence and risk for mortality [51]. *CLEC19A* is a member of the C-type lectin superfamily identified in brain tissues based on the HPA RNA seq project. A literature search revealed few studies which reported the *CLEC19A* but researchers have not treated the role of the *CLEC19A* gene and its protein in much detail [52]. Our bioinformatic results have shown that the CLEC19A protein possesses a signal peptide and no transmembrane domain, which suggests that CLEC19A is a potential secreted protein. Also, confocal microscopy imaging and Western blot analysis indicate that CLEC19A must be a secreted protein that can decrease cell proliferation. our results are in accord with Nauroy and collaborators results. They compared the matrisome proteins of zebrafish, humans, and mouse in an *in-silico* study. They identified 16 genes in human and zebrafish, 6 genes belonged to the core matrisome and 10 genes were matrisome-associated genes. One of the 10 genes has encoded an ECM-affiliated protein (CLEC19A) [53].

According to InterPro server results, the carbohydrate and Ca^2+^ binding potential of CLEC19A protein were investigated. The role of calcium signaling in different cellular functions and various malignancies including cancer has been proved. There is evidence that calcium signaling alteration has a profound impact on cancer development [54, 55]. Based on our study, CLEC19A protein has calcium binding sites and its expression decreased both in mRNA and protein levels in glioma. According to these results, one of leading results of *CLEC19A* reduction in brain cancer can be alteration in extracellular and intracellular calcium concentration.

Our bioinformatic analysis of TCGA data has shown that *CLEC19A* expression in LGG and GBM tissues is significantly decreased compared to normal tissues. Our Experimental analyses have shown that *CLEC19A* notably reduced in glioma tissues and cell lines, verified by *in-silico* data. These data drew our attention to the role of C-lectin members in cancer inhibition such as the negative role of *CLEC3B* in the development of cancers [21], and cell apoptosis promotion due to *CLEC14A* overexpression in lung adenocarcinoma (LUAD) [56]. Given the fact that *CLEC19A* has the highest expression in the normal human brain compared to other human tissues (HPA project) and is expressed at a low level in glioma, we investigate the role of *CLEC19A* and its function in glioma cells through in vitro and in vivo experiments.

Recent studies documented that the activation of the PI3K/AKT/mTOR and PI3K/Akt/NF-κB signaling pathways can promote cell proliferation, cancer metabolism, invasion, and migration [57–59]. Consistent with previous findings, we observed that the overexpression of *CLEC19A* significantly suppressed the proliferative potential of glioma cells through the inhibition of *PI3K*, *AKT1*, and *NF-κB*. Also, our in vitro evidence has shown that the expression level of *PDCD4* and *PTEN* increased in glioma cells after *CLEC19A* overexpression. Together, these data demonstrated that overexpression of the *CLEC19A* gene can reduce cell proliferation and viability through the suppression of the PI3K/Akt/NF-κB signaling pathway.

MMPs, that are associated with metastasis and invasion, are increased in almost all human cancers [60]. Recent studies have highlighted the direct implication of *VEGFα* and MMPs in cell proliferation, migration, invasion, and vascular cell permeability [61, 62]. In agreement with these studies, we showed that overexpression of *CLEC19A* significantly decreased the *VEGFα* gene. Furthermore, we found that *CLEC19A* overexpression reduced *MMP2* activity through increased expression of *TIMP3* and *RECK*. These results suggest that overexpression of *CLEC19A* significantly reduces the potential migration of GBM cancer cells by inhibiting the genes involved in the migration pathway. As expected, our results show that the overexpression of *CLEC19A* promoted cell apoptosis and arrested cells in the sub-G1 phase in U87 and C6 cell lines. *CLEC19A* overexpression could increase the expression of *CDKN1A*, *BAX*, and *BCL2L11* and reduce *BCL2* and *CCNA2* expression. Our results indicated that *CLEC19A* overexpression increased BAX/BCL2 ratio by 3.46-fold compared with the mock group at the protein level. This finding is a significant indicator of an augmented apoptosis rate in cells expressing *CLEC19A*. These results align with previous research that an increase in the expression of *BAX* and *BCL2L11* and a decrease in the level of *BCL2* could lead to apoptosis [63].

Together, these results provide important insights that the overexpression of *CLEC19A* could inhibit cell proliferation, viability, and migration and also induce cell apoptosis in glioma cells. Besides, *CLEC19A* overexpression could arrest the cell cycle in the sub-G1 phase. In regard to these results, some of the members of the C-type lectin superfamily like *CLEC19A* consist of *CLEC10A* [64], *CLEC2* [65], and *CLEC9A* [66] suppressed cell proliferation, migration, and invasion, and also cell cycle arrest, and promoted apoptosis in cancer cells. In contrast, up-regulation of other members from this superfamily such as *CLEC18B* [18], *CLEC5A* [67], *CLEC4A*, and *CLEC4L* [22] were reported to be associated with development, proliferation, migration, and invasion in cancer cells.

To substantiate the in vitro results, in vivo experimentation was carried out in male Wistar rats. We observed that the overexpression of *CLEC19A* significantly decreased brain tumor volume size in the rat model of glioma, which indicated a putative tumor suppressor gene function of the *CLEC19A* gene.

## Conclusions

**Fig. 7:**
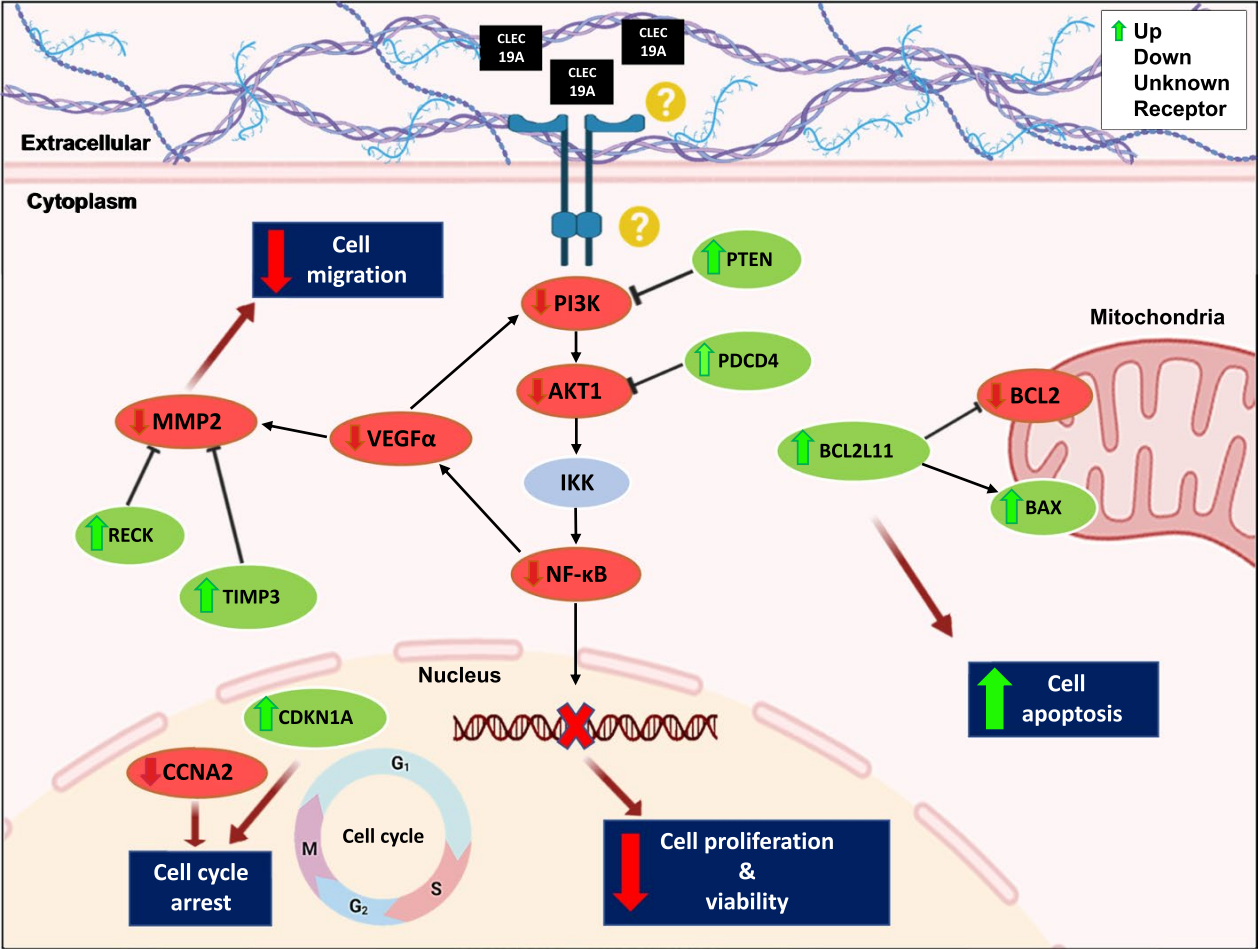
Schematic representation of different pathways regulation by *CLEC19A* overexpression. The overexpression of *CLEC19A*, targeting the *PI3k*, *PTEN*, *AKT*, *NF-κB*, and *PDCD4* transcripts results in reduced PI3K/*AKT*/*NF-κB* signaling pathway activity which affects the biological process of cells, including cell proliferation and viability. Moreover, *CLEC19A* regulates cell migration in this model by targeting VEGFα, *RECK*, *TIMP3*, and *MMP2*. *CLEC19A* overexpression can reduce cell migration in glioma cells. As shown in this Figure, overexpression of *CLEC19A* can promote apoptosis by targeting *BCL2L11*, *BAX*, and *BCL*, and also arrest the cell cycle by reduction of *CCNA2* expression and up-regulation of *CDKN1A* (Parts of the figure are drawn by the BioRender site)

In summary, this study suggests that *CLEC19A* overexpression reduces tumor cell proliferation and migration by suppressing PI3K/AKT/NF-κB and VEGF/MMPs signaling pathways and promotes cell apoptosis and arrested cells in the sub-G1 phase in U87 and C6 cell lines (Fig. 7). Furthermore, overexpression of *CLEC19A* decreased brain tumor volume size in the rat model of glioma. However, our study has a few limitations, including a small sample size due to difficulties recruiting patients and investigating more genes from pathways involved in GBM cancer. It is noteworthy that the other 2 isoforms of CLEC19A need to be investigated. These results enhance our understanding of the role of *CLEC19A* in GBM and may have implications for the development of novel therapeutics to counter GBM. Therefore, further investigation is warranted to identify the exact underlying mechanism behind the role of *CLEC19A* in GBM.

### Supplementary Information

**Additional file 1:** **Fig. S1.** *CLEC19A* structure and its Expression in human normal tissues. (A) CLEC19A is located on 16p12.3 and it has 3 protein-coding variants. (B) The main variant of CLEC19A has 5 exons and the length of this polypeptide is 186 amino acids. (C) The evaluation of CLEC19A expression in different normal tissues. The *CLEC19A* mRNA is abundantly expressed in brain tissue compared to other human normal tissues (www.ncbi.nlm.nih.gov). **Fig. S2.** pCL19A and pCL19A-ORF plasmid structure. (A) CLEC19A cDNA full-length was cloned into the pCDNA3.1(+) vector between the sites of EcoRI and NotI (pCL19A). (B) The ORF fragment of the CLEC19A gene was inserted between the EcoRI and SalI sites of the pEGFP-N1 vector in a frame with the EGFP sequence (pCL19A-ORF). Pictures were created with SnapGene software. **Fig. S3.** 1×106 C6 cells from each group (untreated, Mock, and overexpression construct) were injected into the Caudate Putamen striatum (AP): -2 mm, (ML): 2 mm, and (DV): -4 mm according to the Paxinus Watson atlas, by infusion pump. This image was taken immediately after the injection of C6 cells by MRI (0h). **Fig. S4.** Analysis of CLEC19A with the DeepTMHMM and TOPCONS servers. (A, B) The results of the DeepTMHMM and TOPCONS servers have shown that the CLEC19A protein has an Nterminal signal peptide and no transmembrane domain and it confirms that probably this protein is a secreted protein. (C) CLEC19A contains a signal peptide at its N-terminus from amino acid residues 1 to 19, with a C-type lectin domain from amino acids 40 to 180. Also, it has 2 disulfide bridges. **Fig. S5.** 3 different slices of MRI images for each group. The thickness of each slice is 0.8 millimeters. **Table S1.** The clinicopathological characteristic of patients with glioma cancer. **Table S2.** The list of primer and oligo sequences was used in this study. **Table S3.** Model structures validation for CLEC19A and CLEC19A/GFP fusion protein using Robetta and UCSF ALPHAFOLD2 colab servers.

**Additional file 2.** Original images for Western Blot.

## Data Availability

All real and simulated data used in this paper are publicly available. Our usage of the data is in compliance with the data use agreements of each source. This article contains Supplementary Information. All data can be available upon request from Majid Sadeghizadeh, Tarbiat Modares University, sadeghma@modares.ac.ir, https://www.linkedomics.org/data_download/TCGA-GBM/, https://www.linkedomics.org/data_download/TCGA-LGG/.

## Abbreviations

GBM: Glioblastoma multiforme
LGGs: Low-grade gliomas
WHO: World Health Organization
CTLs: C-type lectins
CLEC19A: C-Type Lectin Domain Containing 19A
CFSE: Carboxyfluorescein succinimidyl ester
TCGA: The Cancer Genome Atlas
NCBI: National Center for Biotechnology Information
ANOVA: One-way analysis of variance
qRT-PCR: Quantitative real-time PCR
NF-κB: Nuclear factor kappa-B
PI3K: Phosphoinositide 3-kinase
TIMP3: Tissue inhibitor of metalloproteinase 3
RECK: Reversion Inducing Cysteine-Rich Protein with Kazal Motifs
MMP2: Matrix metalloproteinase-2
BAX: BCL2 associated X, apoptosis regulator
BCL2: BCL2 apoptosis regulator
PDCD4: Programmed cell death 4
CCNA2: Cyclin A2
BCL2L11: BCL2 like 11
CDKN1A: Cyclin-Dependent Kinase Inhibitor 1A
VEGFA: Vascular Endothelial Growth Factor A
PTEN: Phosphatase and Tensin Homolog
