# Supplementary material for: CLEC19A overexpression inhibits tumor cell proliferation/migration and promotes apoptosis concomitant suppression of PI3K/AKT/NF-κB signaling pathway in glioblastoma multiforme
Source: BMC Cancer. 2024 Jan 2;24:19. doi: 10.1186/s12885-023-11755-9 (PMC10763001; doi:10.1186/s12885-023-11755-9)
Supplement: Supplementary file 1 — Additional file 1: Fig. S1. CLEC19A structure and its Expression in human normal tissues. (A) CLEC19A is located on 16p12.3 and it has 3 protein-coding variants. (B) The main variant of CLEC19A has 5 exons and the length of this polypeptide is 186 amino acids. (C) The evaluation of CLEC19A expression in different normal tissues. The CLEC19A mRNA is abundantly expressed in brain tissue compared to other human normal tissues (www.ncbi.nlm.nih.gov). Fig. S2. pCL19A and pCL19A-ORF plasmid structure. (A) CLEC19A cDNA full-length was cloned into the pCDNA3.1(+) vector between the sites of EcoRI and NotI (pCL19A). (B) The ORF fragment of the CLEC19A gene was inserted between the EcoRI and SalI sites of the pEGFP-N1 vector in a frame with the EGFP sequence (pCL19A-ORF). Pictures were created with SnapGene software. Fig. S3. 1×10^6^ C6 cells from each group (untreated, Mock, and overexpression construct) were injected into the Caudate Putamen striatum (AP): -2 mm, (ML): 2 mm, and (DV): -4 mm according to the Paxinus Watson atlas, by infusion pump. This image was taken immediately after the injection of C6 cells by MRI (0h). Fig. S4. Analysis of CLEC19A with the DeepTMHMM and TOPCONS servers. (A, B) The results of the DeepTMHMM and TOPCONS servers have shown that the CLEC19A protein has an Nterminal signal peptide and no transmembrane domain and it confirms that probably this protein is a secreted protein. (C) CLEC19A contains a signal peptide at its N-terminus from amino acid residues 1 to 19, with a C-type lectin domain from amino acids 40 to 180. Also, it has 2 disulfide bridges. Fig. S5. 3 different slices of MRI images for each group. The thickness of each slice is 0.8 millimeters. Table S1. The clinicopathological characteristic of patients with glioma cancer. Table S2. The list of primer and oligo sequences was used in this study. Table S3. Model structures validation for CLEC19A and CLEC19A/GFP fusion protein using Robetta and UCSF ALPHAFOLD2 colab servers. [file 12885_2023_11755_MOESM1_ESM.pdf]

Figure S1

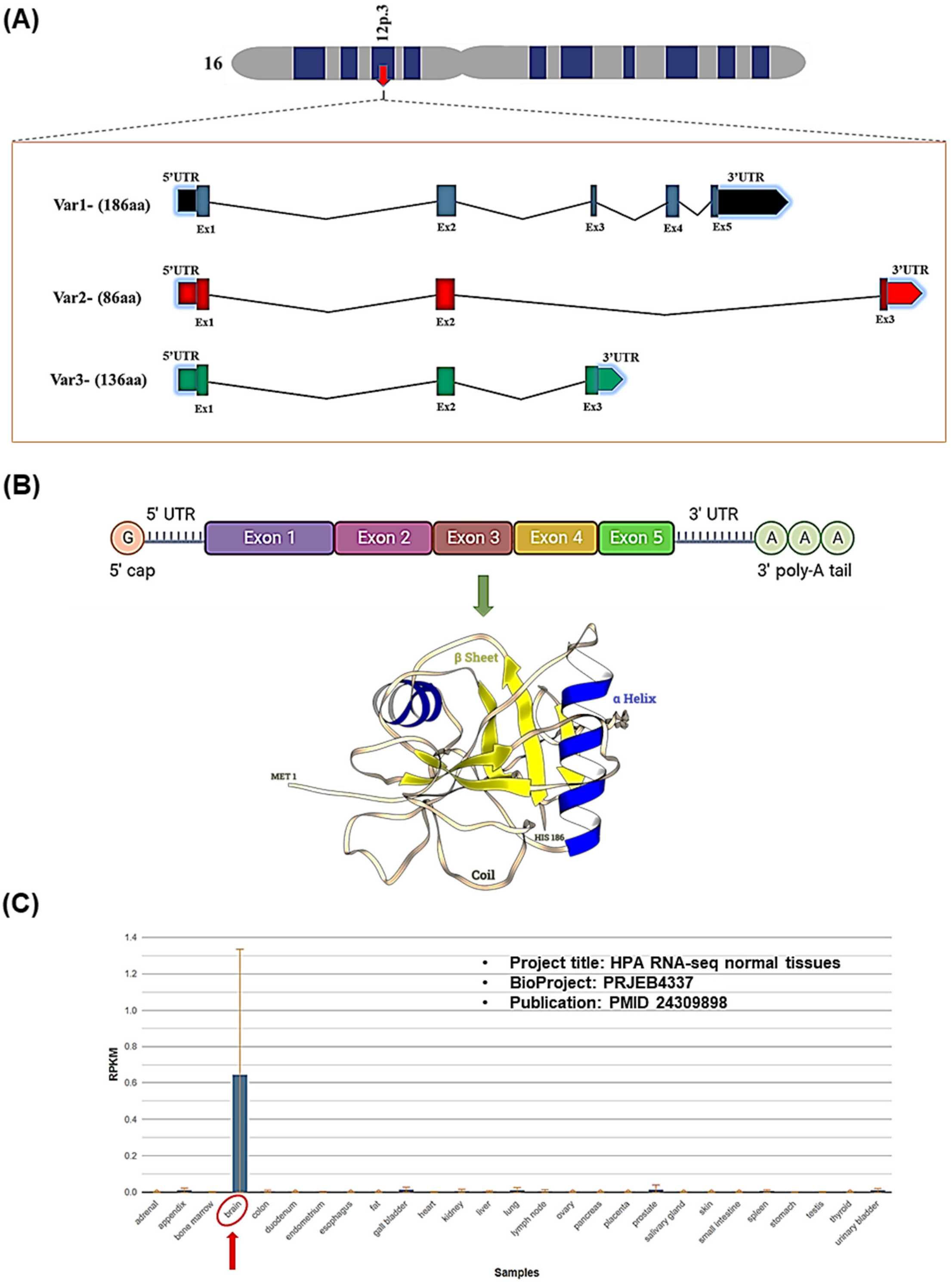

Fig. S1. *CLEC19A* structure and its Expression in human normal tissues. (A) CLEC19A is located on 16p12.3 and it has 3 protein-coding variants. (B) The main variant of CLEC19A has 5 exons and the length of this polypeptide is 186 amino acids. (C) The evaluation of *CLEC19A* expression in different normal tissues. The *CLEC19A* mRNA is abundantly expressed in brain tissue compared to other human normal tissues (www.ncbi.nlm.nih.gov).

Figure S2

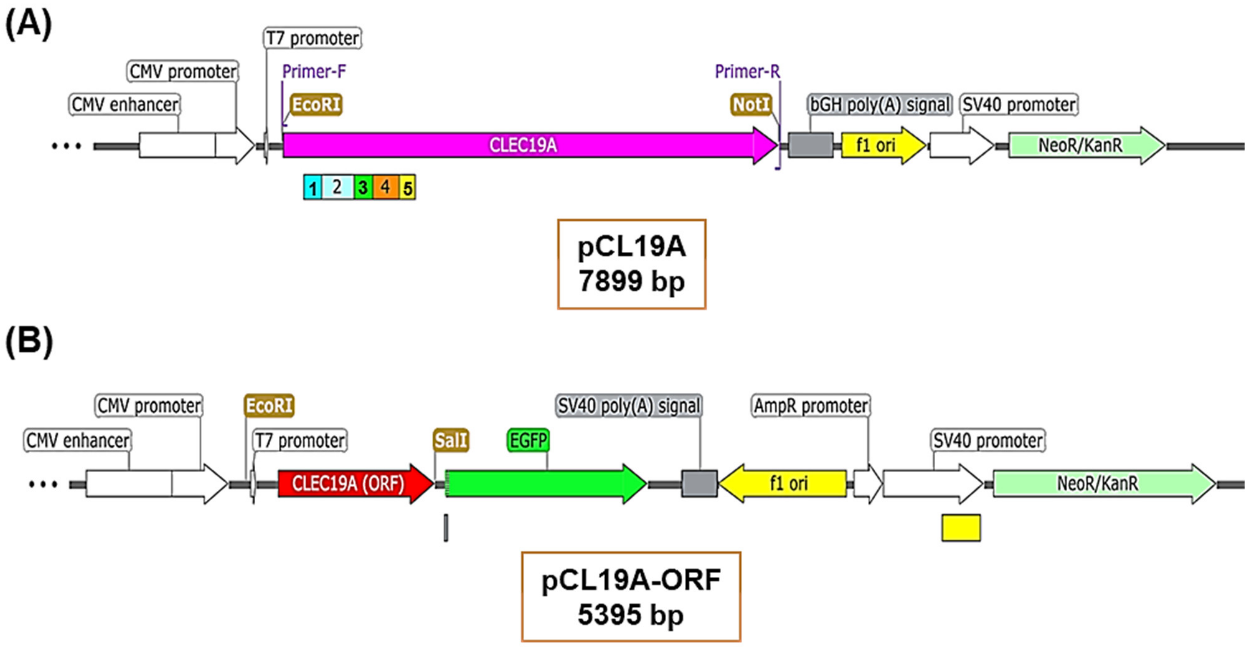

Fig. S2. pCL19A and pCL19A-ORF plasmid structure. (A) *CLEC19A* cDNA full-length was cloned into the pCDNA3.1(+) vector between the sites of EcoRI and NotI (pCL19A). (B) The ORF fragment of the *CLEC19A* gene was inserted between the EcoRI and SalI sites of the pEGFP-N1 vector in a frame with the EGFP sequence (pCL19A-ORF). Pictures were created with SnapGene software.

Figure S3

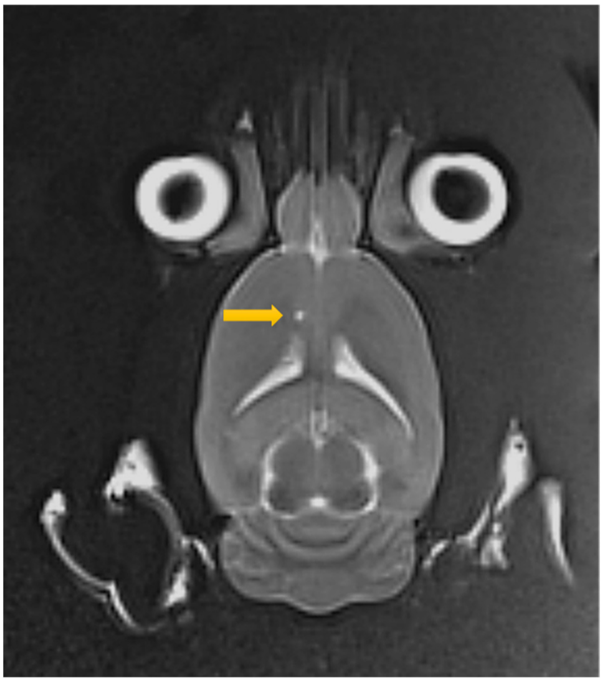

Fig. S3. 1×106 C6 cells from each group (untreated, Mock, and overexpression construct) were injected into the Caudate Putamen striatum (AP): -2 mm, (ML): 2 mm, and (DV): -4 mm according to the Paxinus Watson atlas, by infusion pump. This image was taken immediately after the injection of C6 cells by MRI (0h).

Figure S4

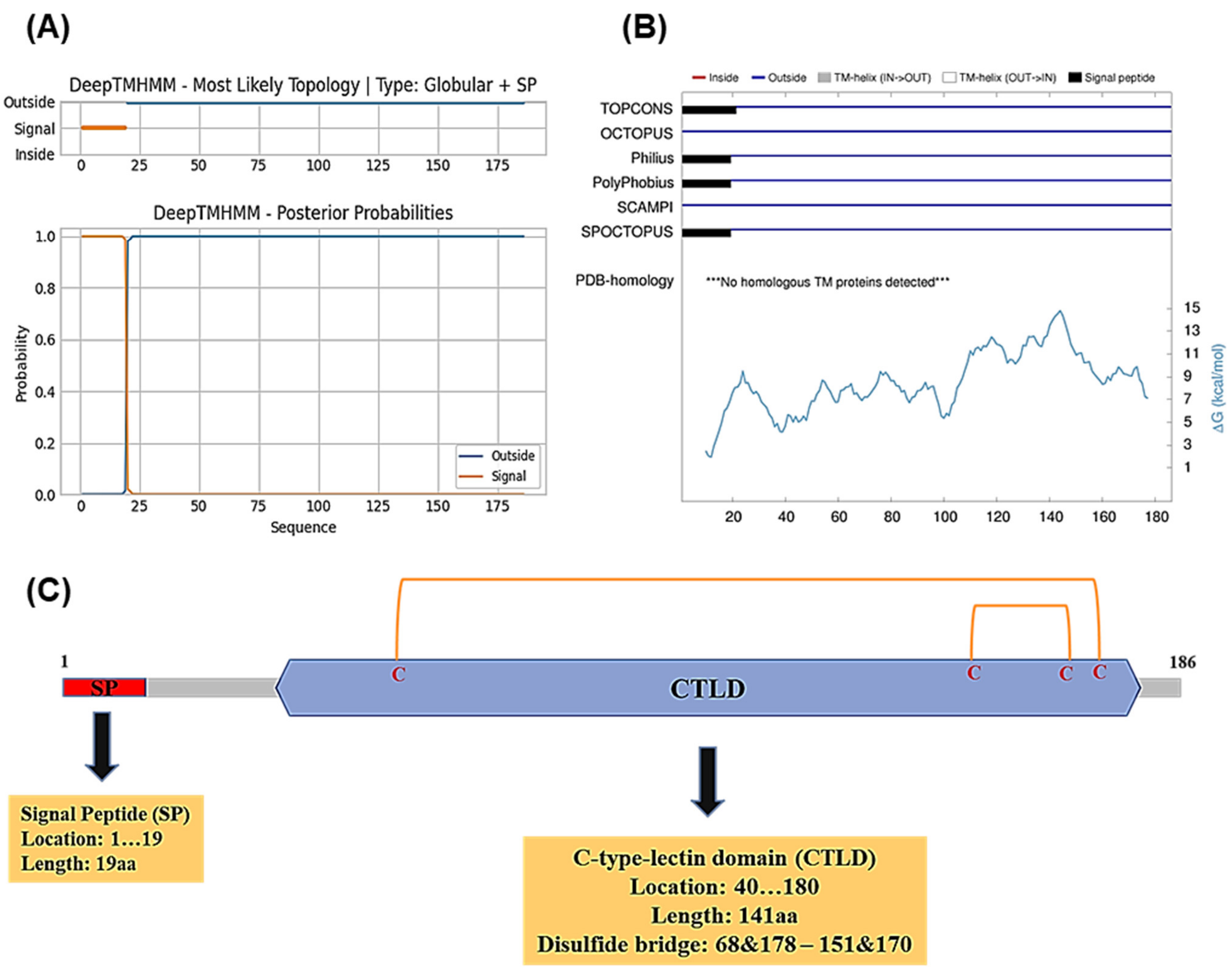

Fig. S4. Analysis of CLEC19A with the DeepTMHMM and TOPCONS servers. (A, B) The results of the DeepTMHMM and TOPCONS servers have shown that the CLEC19A protein has an N-terminal signal peptide and no transmembrane domain and it confirms that probably this protein is a secreted protein. (C) CLEC19A contains a signal peptide at its N-terminus from amino acid residues 1 to 19, with a C-type lectin domain from amino acids 40 to 180. Also, it has 2 disulfide bridges.

Figure S5

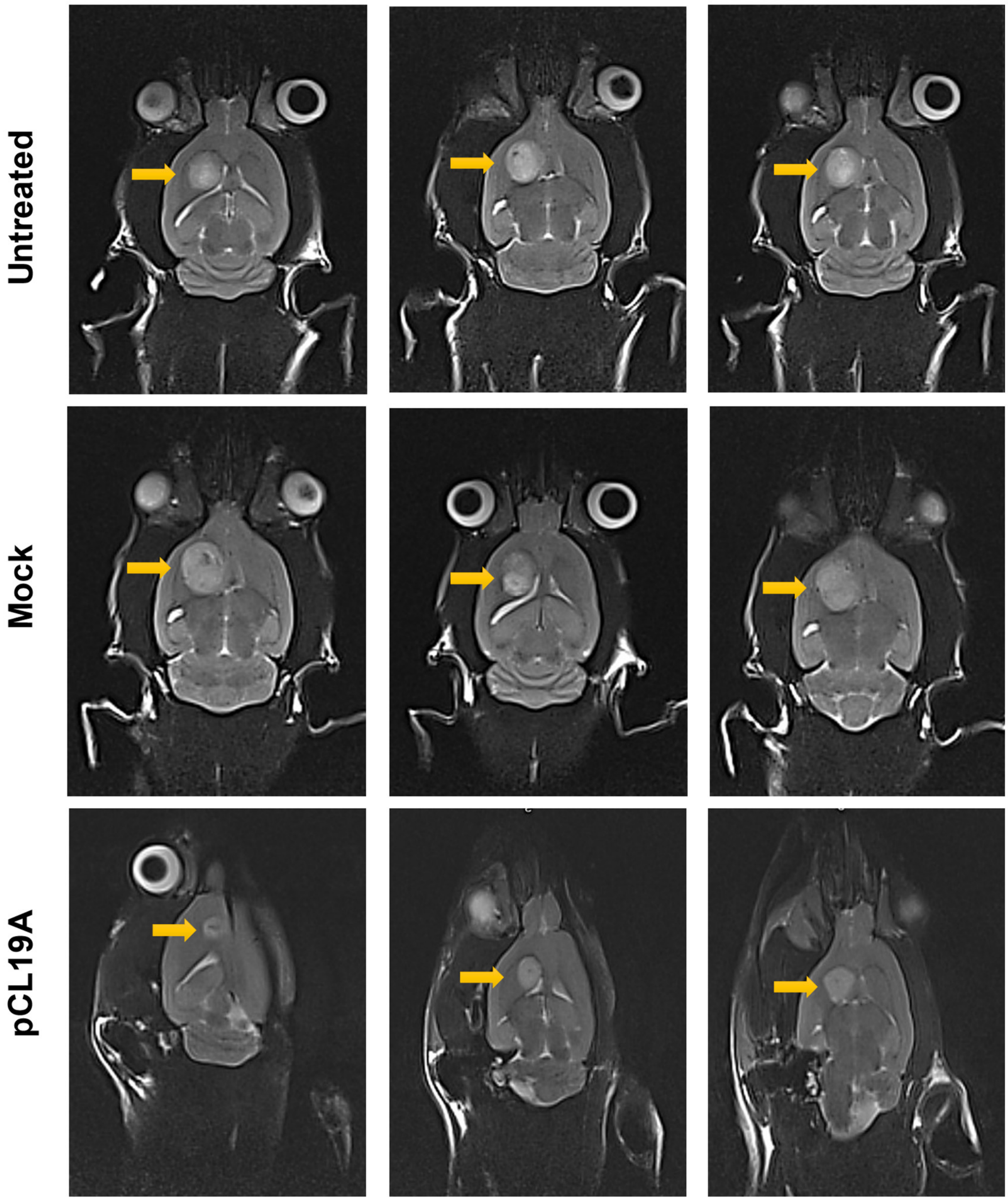

Fig. S5. 3 different slices of MRI images for each group. The thickness of each slice is 0.8 millimeters.

Table S1

No	Sex	Age	Type	Grade	Location
1	Male	35	LGG	II	Left temporal lobe
2	Male	36	LGG	II	Left frontal lobe
3	Male	54	LGG	II	Right frontal lobe
4	Female	63	LGG	II	Left temporal occipital lobe
5	Male	57	LGG	II	Right temporal parietal lobe
6	Male	42	LGG	II	Left temporal lobe
7	Male	50	LGG	II	Right parietal temporal lobe
8	Female	60	LGG	II	Right occipital lobe
9	Male	36	Normal (Edge of tumor-patient 2)	-	Left frontal lobe
10	Male	54	Normal (Edge of tumor-patient 3)	-	Right frontal lobe
11	Male	42	Normal (Edge of tumor-patient 6)	-	Left temporal lobe
12	Female	60	Normal (Edge of tumor-patient 8)	-	Right occipital lobe
13	Male	68	GBM	IV	Left parietal lobe
14	Male	53	GBM	IV	Right frontal temporal lobe
15	Male	66	GBM	IV	Left temporal lobe
16	Male	49	GBM	IV	Left frontal temporal lobe
17	Female	62	GBM	IV	Right frontal lobe
18	Male	41	GBM	IV	Left parietal lobe
19	Male	56	GBM	IV	Left temporal lobe and Cerebellum

Table S1. The clinicopathological characteristic of patients with glioma cancer.

Table S2

Sequences of primers			
**Gene**	**Primer (5'>3')**	**Size (bp)**	**Size of PCR Product (bp)**
Forward primer for CLEC19A-M	GTCTGGACAGGCCTTCATGATC	22	182
Reverse primer for CLEC19A-M	TCATTCCATGACCTCAGAGCAC	22	
Forward primer for rClec19a	TGTTCCCAGCACTCAAGTCCT	21	91
Reverse primer for rClec19a	CTGTGCAAGTCTAGGGCGG	19	
Forward primer for β-actin	GCCAGCTCACCATGGATGATG	21	158
Reverse primer for β-actin	CTGACCCATGCCCACCATCACGCC	24	
Forward primer for rGapdh	TGTGACTTCAACAGCAACTCCCAT	24	206
Reverse primer for rGapdh	CTCTCTTGCTCTCAGTATCCTTGC	24	
Forward primer for MMP2	TCCCAGCGGCCAAAGTTGATC	21	179
Reverse primer for MMP2	AAGTGGGACAAGAACCAGATC	21	
Forward primer for RECK	CATCACACAAACTGCCGAGAA	21	96
Reverse primer for RECK	GGCGCAATAATTTTCCACTGCT	22	
Forward primer for TIMP3	GTGCAACTTCGTGGAGAGGT	20	109
Reverse primer for TIMP3	CAGGTAGTAGCAGGACTTGATCTTG	25	
Forward primer for VEGFα	GTGGTGAAGTTCATGGATGTCTA	23	115
Reverse primer for VEGFα	CACAGGATGGCTTGAAGATGTA	22	
Forward primer for rVegfα	CTCTGCTAATGTTATTGGTGTCTTC	25	230
Reverse primer for rVegfα	ATGGGTTTGTCGTGTTTCTGG	21	
Forward primer for PI3K	GGTGAAAGACGATGGACAACTG	22	191
Reverse primer for PI3K	TGTAACACATCTCCTGAAACCTCTC	25	
Forward primer for PTEN	CACACGACGGGAAGACAAGT	20	162
Reverse primer for PTEN	TCCTCTGGTCCTGGTATGAAG	21	
Forward primer for AKT1	AACGGGGGCGAGCTGTTC	18	115
Reverse primer for AKT1	TCTTCTCCGAGTGCAGGTAGTCC	23	
Forward primer for rAkt1	ACGACGTAGCCATTGTGAAG	20	226
Reverse primer for rAkt1	CAGCGGATGATAAAGGTGTTG	21	
Forward primer for PDCD4	CAGTTGGTGGGCCAGTTTATTG	22	131
Reverse primer for PDCD4	AGAAGCACGGTAGCCTTATCCA	22	
Forward primer for NF-κB	TCTTACACTTAGCAATCATCCAC	23	178
Reverse primer for NF-κB	CCAGCCCTCAGCAAATCCTCC	21	
Forward primer for BAX	AGAGGATGATTGCCGCCGTG	20	117
Reverse primer for BAX	AGTAGAAAAGGGCGACAACCC	21	
Forward primer for rBax	CAGAGGATGATTGCTGACG	19	116
Reverse primer for rBax	TAGAAGAGGGCAACCAC	17	
Forward primer for BCL2L11 (BIM)	CAAGTCAACACAAACCCCAAGTC	23	74
Reverse primer for BCL2L11 (BIM)	GTCGTATGGAAGCCATTGCA	20	
Forward primer for BCL2	CCGAGATGTCCAGCCAGCTG	20	119
Reverse primer for BCL2	CTCAAAGAAGGCCACAATC	19	
Forward primer for CCNA2 (Cyclin A2)	CAATGGATGGTAGTTTTGAGTCAC	24	215
Reverse primer for CCNA2 (Cyclin A2)	TAACCAGTCCACGAGGATAGC	21	
Forward primer for CDKN1A (P21)	TCTTGTACCCTTGTGCCTC	19	111
Reverse primer for CDKN1A (P21)	GTGGTAGAAACTCGTCATGC	20	
Forward primer for rCdkn1a (rP21)	GTGATATGTACCAGCCACAGGC	22	260
Reverse primer for rCdkn1a (rP21)	GGCTCAGGTAGATCTTGGGCA	21	
Forward primer for CLEC19A-C	CTGGGAGGTTGCTTTCTAGGAG	22	587
Reverse primer for GFP-SalI	CACCGTCGACTGAATGGTCAGAGATGGGATTTTGC	35	
Forward primer for CLEC19A-EcoRI	CCAACGAATTCCAGGCTCCATCTGACCCTAG	31	2491
Reverse primer for CLEC19A-NotI	AATTAAGCGGCCGCATGTACAGTTCAGTGGCTTCTTG	37	
Forward primer for M13	CTCGGTACCGTAATACGACTCAC	23	189
Reverse primer for M13	GGAAACAGCTATGACCATGATTACGC	26	
Forward primer for GFP	CAGAAGAACGGCATCAAGGTG	21	140
Reverse primer for GFP	GTGCTCAGGTAGTGGTTGTCG	21	
Forward primer for pCDNA3.1+	TATAGGGAGACCCAAGCTGGCTAGC	25	161
Reverse primer for pCDNA3.1+	AAGGCACAGTCGAGGCTGATC	21	

Table S2. The list of primer and oligo sequences was used in this study.

Table S3

Name	Modeling server	Model	PROCHECK (%)	ERRAT (%)
**CLEC19A**	UCSF Alphafold2 colab	1	66	53.57
		2	71	57.41
		3	68.5	53.25
		4	69.8	61.44
		5	71	66.03
	Robetta	1	82.3	73.75
		2	82.3	80.71
		3	82.3	73.75
		4	84.7	**81.69**
		5	86.9	81.42
**CLEC19A/GFP**	UCSF Alphafold2 colab	1	78	77.02
		2	80.2	**81.79**
		3	80.7	81.29
		4	79.9	78
		5	81.5	75.71
	Robetta	1	85.4	73.91
		2	85.6	74.28
		3	86.2	66.66
		4	86.9	81.32

Table S3. Model structures validation for CLEC19A and CLEC19A/GFP fusion protein using Robetta and UCSF ALPHAFOLD2 colab servers.
