# Supplementary figures and images for: CLEC19A overexpression inhibits tumor cell proliferation/migration and promotes apoptosis concomitant suppression of PI3K/AKT/NF-κB signaling pathway in glioblastoma multiforme
Source: BMC Cancer. 2024 Jan 2;24:19. doi: 10.1186/s12885-023-11755-9 (PMC10763001; doi:10.1186/s12885-023-11755-9)

Original images for Western Blot

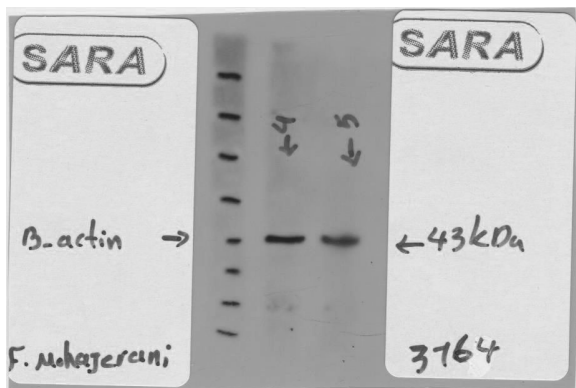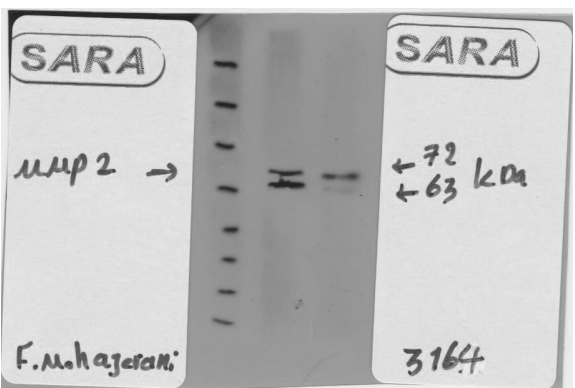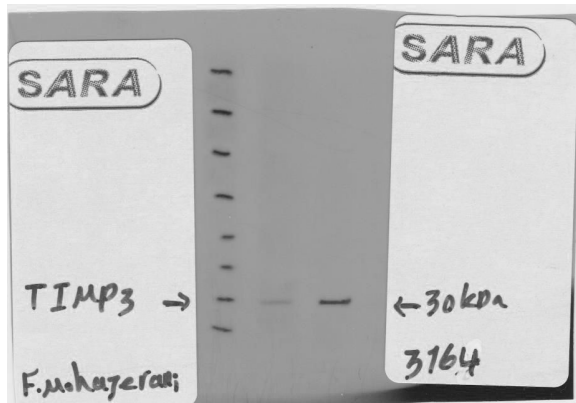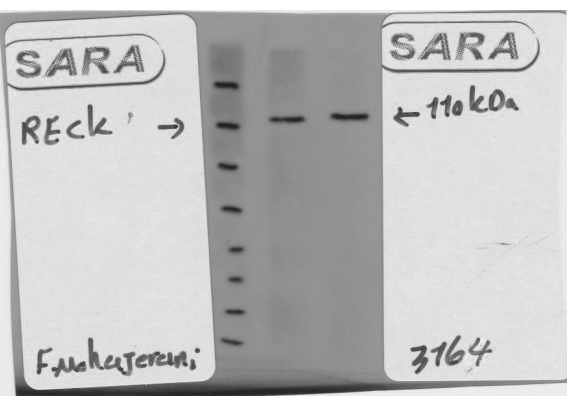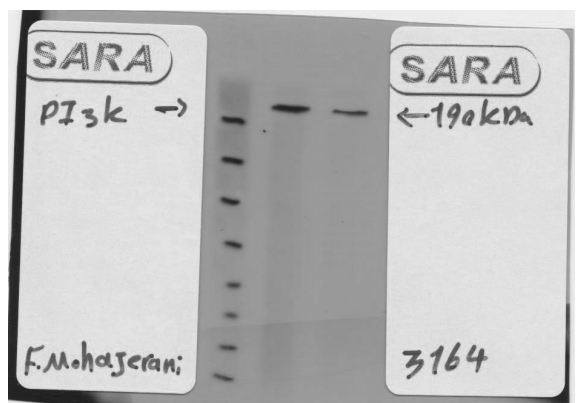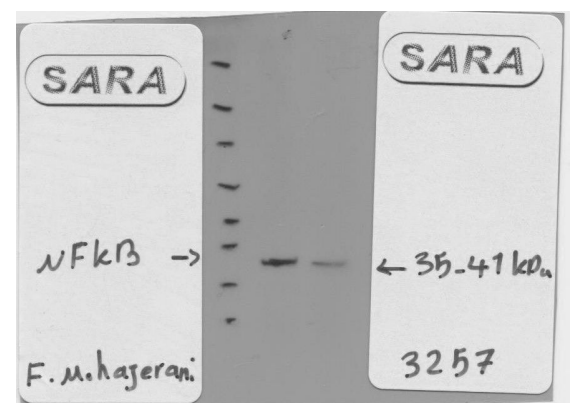

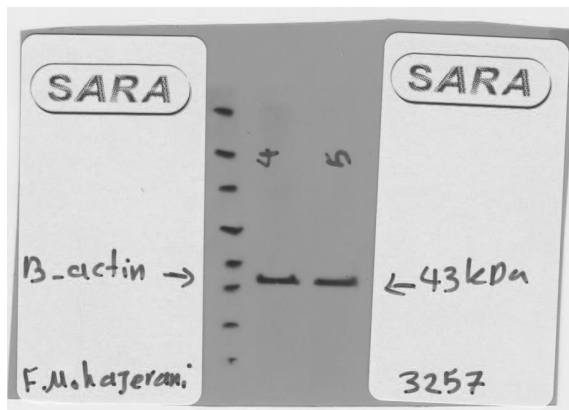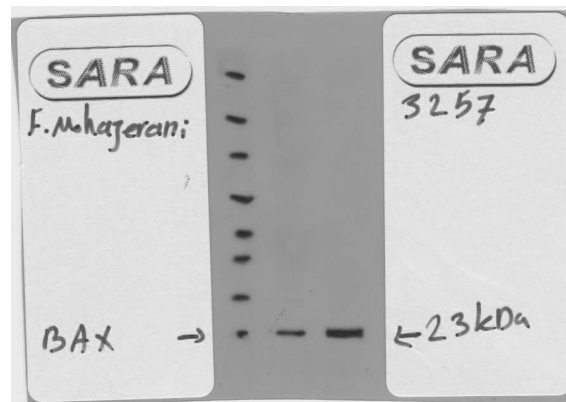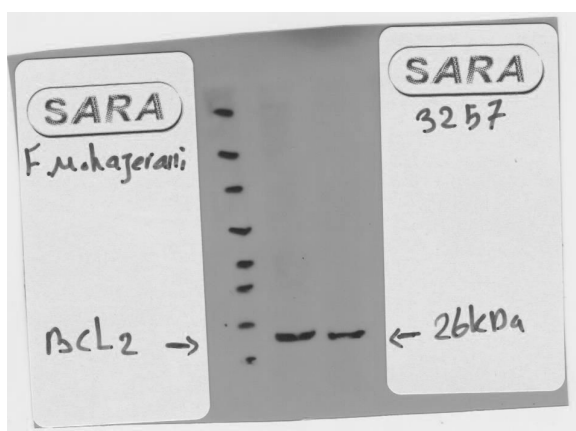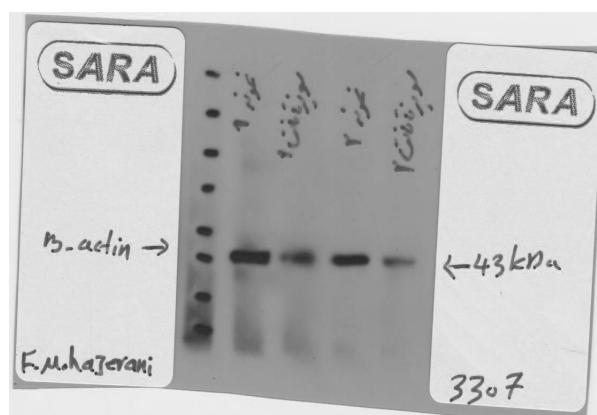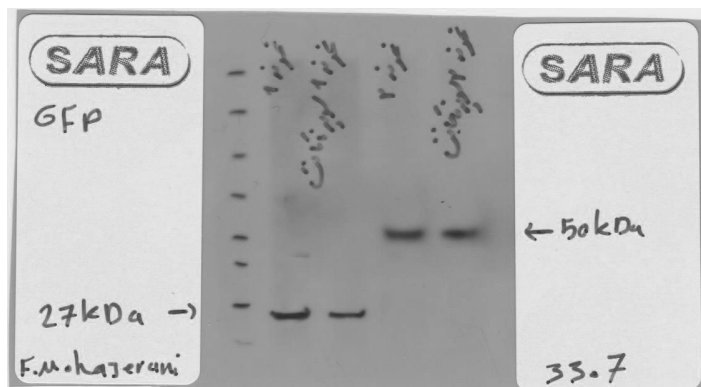

Supplement: Supplementary file 2 — Additional file 2. Original images for Western Blot. [file 12885_2023_11755_MOESM2_ESM.pdf]
